# On the Use of Nudges to Affect Spillovers in Environmental Behaviors

**DOI:** 10.3389/fpsyg.2019.00061

**Published:** 2019-01-29

**Authors:** Valeria Fanghella, Giovanna d’Adda, Massimo Tavoni

**Affiliations:** ^1^Department of Economics and Management, University of Trento, Trento, Italy; ^2^Department of Economics, Management, and Quantitative Methods, University of Milan, Milan, Italy; ^3^Department of Management, Economics and Industrial Engineering, Politecnico di Milano, Milan, Italy; ^4^RFF-CMCC European Institute on Economics and the Environment (EIEE), Fondazione CMCC, Lecce, Italy

**Keywords:** spillover effect, moral licensing, environmental identity, social information, goal commitment, online experiment

## Abstract

Environmental self-identity is considered a promising lever to generate positive spillovers across pro-environmental behaviors: existing evidence shows that it is positively correlated with pro-environmental choices and that it can be easily manipulated, by reminding individuals of their past pro-environmental actions. However, it remains unclear whether it can be successfully used for environmental policy making. In two online, incentive-compatible experiments, we manipulate participants’ environmental self-identity and test whether this leads to increased donations to an environmental charity. Additionally, we investigate the interaction between self-identity priming and two commonly used behavioral policy tools: social information (Study 1, *N* = 400) and goal commitment (Study 2, *N* = 495). Our results suggest caution in leveraging environmental self-identity to promote pro-environmental behaviors, provide indications on how to target policies based on self-identity primes, and offer novel evidence on the interaction between different behavioral policy tools.

## Introduction and Hypothesis

Promoting pro-environmental behavior in individuals and organizations is key to addressing global environmental threats such as climate change, air pollution, and resource depletion. Academics and policymakers have tested a variety of instruments to induce people to behave more environmentally, ranging from traditional policy tools, like incentives and regulation, to softer behavioral interventions, like information provision and nudging. Evaluation of these policies must crucially keep into account not only their direct impact, but also their spillover effects on other pro-environmental behaviors (Truelove et al., 2014; Dolan and Galizzi, 2015; d’Adda et al., 2017; Ghesla et al., 2018; Schmitz, 2018). The overall impact of environmental policies will be positive only in so far that any direct effect, that they may have, will not be offset by compensating behaviors, either in other domains or for the same activity over time. In designing effective policies, regulators therefore need to know whether encouraging people to act pro-environmentally will generate positive or negative spillovers over time or in other domains.

Acting pro-environmentally is likely to generate positive returns in terms of self and social image (Mazar et al., 2008; Ariely et al., 2009; Gneezy et al., 2012). But what is the impact of positive self and social image on subsequent pro-environmental conduct? A related concept is environmental self-identity, defined as the extent, to which people see themselves as someone who behaves pro-environmentally (Van der Werff et al., 2014b). Environmental self-identity has been found to significantly correlate with pro-environmental behavior in a widespread set of domains, such as water and energy conservation, waste reduction, sustainable shopping, transportation and environmental activism (Cook et al., 2002; Clayton and Opotow, 2003; Fielding et al., 2008; Nigbur et al., 2010; Whitmarsh and O’Neill, 2010; Van der Werff et al., 2013b; Gatersleben et al., 2014; Peters et al., 2018).

Beyond a stable core that directly depends on values, identity can be manipulated to some extent. Namely, by reminding individuals of their past pro-environmental behaviors, it is possible to strengthen environmental self-identity (Cornelissen et al., 2008; Van der Werff et al., 2013a, 2014a,b). This methodology is grounded on self-perception theory, which states that “individuals come to know their own internal states by inferring them from observations of their own overt behavior” (Bem, 1972, p. 2). Implied by this method is the presence of positive spillover effects: having engaged in past pro-environmental behaviors increases the likelihood that one will behave pro-environmentally also in the future. Hence, self-identity theories suggest not only that policies inducing pro-environmental acts will generate positive spillover effects through their impact on individuals’ environmental self-identity; but also that environmental self-identity primes should be included in the design of environmental campaigns, as they may encourage many different pro-environmental actions. Given that self-identity can be activated by means of situational cues, it would be a simple and inexpensive component of policies aimed at fostering individuals to behave pro-environmentally. For instance, Susewind and Hoelzl (2014) suggest leveraging past commitment to environmental activities in the design of fundraising campaigns. Similarly, Van der Werff et al. (2014a) argue that environmental policies could encourage consistency by placing billboards, commercials or reminders of previous engagement in pro-environmental deeds close to places where people make new environmental decisions.

Past moral actions have, however, also been found to discourage subsequent pro-environmental behaviors. In the environmental domain, negative spillover effects have been documented in water and energy consumption, purchase of green products, and cooperative decision-making (Thøgersen and Ölander, 2003; Sachdeva et al., 2009; Mazar and Zhong, 2010; Tiefenbeck et al., 2013). Negative spillover effects have also been detected by many studies on moral and prosocial behavior more in general, including charity support, blood donation, volunteering, and purchasing decisions (Strahilevitz and Myers, 1998; Monin and Miller, 2001; Khan and Dhar, 2006; Jordan et al., 2011; Merritt et al., 2010, 2012; Clot et al., 2016). One of the main explanations of the occurrence of negative spillovers is the moral credit model (Sachdeva et al., 2009), which suggests that individuals establish a moral self-image throughout their lifespan. Hence, they perform compensatory reasoning and actions (Zhong et al., 2009; Miller and Effron, 2010; Merritt et al., 2010; Jordan et al., 2011; Truelove et al., 2014): when engaging in what is commonly perceived as a moral or ethical action, individuals experience an enhanced sense of morality, which provides them with moral credits. Such credits serve to offset subsequent immoral behaviors – namely, moral licensing. In the same way, individuals act more morally when their moral self has been threatened by past immoral conduct – namely, moral cleansing.

In summary, evidence and psychological explanations account for past moral behaviors increasing, as well as decreasing, future moral striving. Reconciling these two sets of evidence requires, in our opinion, to compare the costs associated with the moral action with the psychological costs of behavioral inconsistency. Pro-environmental and moral behaviors entail personal costs, which decrease the attractiveness of moral alternatives (Van der Werff et al., 2013a; Steg et al., 2014). The literature on behavioral consistency shows that manipulating the salience of past pro-environmental decisions can increase the psychological costs of acting inconsistently in subsequent decisions (Festinger, 1962; Fishbach et al., 2006; Guadagno et al., 2001; Thøgersen, 2004). The perception of the target behavior also features in this process of costs evaluation: evidence shows that, if the behavior is relatively unimportant to one’s moral self, past moral deeds are more likely to provide moral credits rather than incentivizing behavioral consistency (Thøgersen, 2004; Thøgersen and Crompton, 2009; Miller and Effron, 2010; Peters et al., 2018). Therefore, if past behaviors are central to the self, the discomfort of acting inconsistently can loom larger than the costs of behaving pro-environmentally.

The current work aims at testing the sign of spillover effects from self-identity priming with a heterogeneous sample and in an incentive compatible way. We conduct two studies with subjects recruited from an online labor platform.^1^ In both studies, we observe how priming environmental self-identity affects subsequent costly donation decisions to an environmental NGO, and investigate sources of heterogeneity in participants’ reaction to priming. We investigate the impact on the sign and magnitude of spillovers of combining self-identity priming with social information (Study 1), and goal commitment (Study 2). We select these two nudges not only because they are among the most popular behavioral policies, but also because existing theories point to social information and goal commitment as two potential levers capable of offsetting moral licensing. As for social information, others’ social behavior can signal one’s moral incompleteness or can correct the misperception of unbalanced contribution to the common cause (Kahneman et al., 1993; Guagnano et al., 1994; Thøgersen and Crompton, 2009; Jordan et al., 2011). Goal commitment shapes induces individuals to interpret previous behaviors as evidence of commitment toward an overarching goal, and motivates them to persist in its attainment (Dhar and Simonson, 1999; Shah et al., 2002; Fishbach et al., 2006; Fishbach et al., 2009; Mullen and Monin, 2016).

In both studies, priming self-identity does not result in positive spillovers. Rather, individuals who are more used to perform pro-environmental behaviors are not affected by the priming, whereas remaining subjects display consistency in failing to act pro-environmentally and display negative cross-behavioral spillovers. Finally, we observe differences in the ability of different nudges to offset the undesired behavior: social information offsets the negative spillovers, whereas goal commitment amplifies them.

Our study makes two main contributions to the literature. First, we provide clean evidence on the impact of environmental identity priming on incentivized behavior. Previous studies reporting positive spillovers from reminding individuals of their past pro-environmental behaviors mainly relied on self-reported measures (Cornelissen et al., 2008; Van der Werff et al., 2013a, 2014a,b), or behaviors that required little or no effort or cost (Cornelissen et al., 2008; Van der Werff et al., 2014b). Given our view that consistency with one’s moral self is a matter of balancing the psychological costs of behavioral inconsistency against the costs of behaving morally, identifying behavioral outcomes that are both directly observable and costly appears critical for testing rigorously and meaningfully the sign of spillovers. We can thus investigate whether the sign of spillover effects differs between our studies and previous ones using less demanding or self-reported tasks as outcomes.

Second, we complement self-identity priming with other common behavioral measures. As behavioral interventions become increasingly popular, individuals are likely to be subject to multiple nudges. However, so far little research exists on the combined effect of different behavioral interventions (Brandon et al., 2018). Since nudges leverage on individuals’ psychology and behavioral fallacies, policy makers should pay attention to the unintended interplays that can occur between the different tools. Indeed, our results illustrate that goal commitment, a behavioral policy that is commonly recommended to prevent moral licensing (Fishbach and Dhar, 2005; Fishbach et al., 2006; Mukhopadhyay et al., 2008), does not achieve the same effect when implemented together with identity priming. Moreover, we identify an innovative strategy to tackle negative spillover effects: in spite of the overarching evidence that social influence affects individuals’ behaviors in a widespread range of domains, such as waste prevention, energy and water saving, towel reuse in hotels, and technology adoption (e.g., Schultz, 1999; Schultz et al., 2007; Goldstein et al., 2008; Nolan et al., 2008; Allcott, 2011; Ferraro et al., 2011; Nomura et al., 2011; Toelch et al., 2011; Harries et al., 2013), to our knowledge, it has not yet been implemented as way to prevent negative spillover. Not only we prove that social information effectively addresses their occurrence, but also provide preliminary evidence on why this happens. Our findings suggest that the negative effect resulting from identity manipulation is likely to be caused by contribution ethic, whereby one refrains from a moral action because of the perception of having “already done one’s own fair share” (Kahneman et al., 1993; Guagnano et al., 1994; Thøgersen and Crompton, 2009). Therefore, providing the information that also other individuals contribute to the common good alleviates this feeling and allows to offsets the negative spillover.

The remainder of the paper proceeds as follows. We describe the experimental design and the results of Study 1 in Section 2, and of Study 2 in Section 3. Section “General Discussion” proposes a discussion of the findings of the two studies, and their implications. Section “Conclusion” concludes.

## Study 1

### Materials and Methods

#### Participants and Procedure

We recruited respondents on the online platform Prolific Academic, a United Kingdom platform giving access to a predominantly European pool of users. In total, 397 subjects completed the experiment. Each participant received a participation fee of £1 and could earn up to £1 as an additional bonus, depending on her decision within the experiment. Namely, subjects decided how much of the £1 bonus, if any, to donate to an environmental organization. The donation was then deducted from the bonus when computing participants’ final payoff. The decision to donate and the donation amount are our main outcome variables.

**FIGURE 1:**
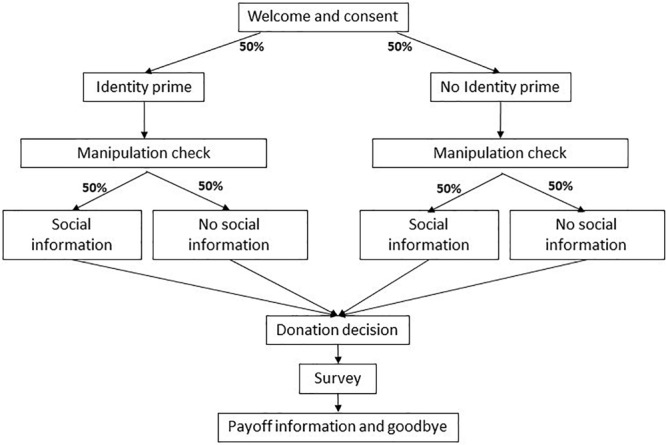
Study 1 experimental protocol.

Before making the donation decision, subjects were randomly assigned to experimental conditions in a two (identity priming versus control) by two (social information versus control) between subjects design. Thus, the four experimental conditions allow to observe, relative to the control group, the impact of providing the identity prime and social information in isolation, and combined. Namely, we first assigned half of the subjects to receive the identity prime. Immediately after, we measured their environmental self-identity to perform a manipulation check, i.e., to test that the prime indeed had the intended effect. Next, half of the subjects from both the identity prime and control groups were randomly assigned to the social information treatment. Only then, all subjects made the donation decision. The experiment ended with a brief survey, including questions on environmental values. The last screen provided information on subjects’ payoff and on how to receive it. Figure 1 summarizes the experimental protocol for Study 1, which is reported in full in the Supplementary Material available online.

#### Materials

##### Treatment 1: identity priming

In order to prime environmental self-identity, we followed the methodology introduced by Cornelissen et al. (2008). The adoption of this priming method has been found to be correlated with higher levels of self-reported environmental self-identity among experimental subjects (Van der Werff et al., 2013a, 2014b). Specifically, we primed environmental self-identity by asking subjects how frequently they engaged in eight pro-environmental behaviors. Answers ranged on a 5-point scale between “Never” and “Always.” The pro-environmental behaviors included in the priming exercise must be common across different countries, so that most subjects in our sample would infer a positive self-identity from their own affirmative answers to the priming questions. We thus selected the behaviors to be included in the priming exercise among the ones most commonly performed by respondents in a series of international studies, namely from Belgium (Cornelissen et al., 2008), the Netherlands (Van der Werff et al., 2013a, 2014b) and United States (Gallup, 2010). Table 1 reports the resulting set of eight actions and the average frequency of engagement among participants assigned to the priming treatment: the actions span a broad range of settings, from energy saving, to recycling, to transport and purchasing choices, and are indeed frequently performed by participants assigned to the identity prime conditions.

**Table 1:** Actions included in the environmental priming exercise and frequency of reported engagement, Study 1.

Action	*M*	*SD*
I turn off the lights when no one is in the room	4.322	0.869
I do not throw litter on the street	4.573	0.966
I recycle newspapers, glass, aluminum, motor oil, or other items	3.794	1.190
I turn off electrical appliances (to save energy)	3.834	1.043
I move around by bike and/or public transportation	3.216	1.359
I buy a less polluting product if there is a choice in the shop	3.095	1.157
I use reusable shopping bags at grocery stores instead of the standard plastic or paper bags	3.769	0.653
I leave a clean spot after a picnic	4.653	0.762
Total	3.907	0.622
Number of observations: identity priming and identity priming plus social information	203	

Participants assigned to the *No identity prime* conditions had to report how often they performed a different set of eight behaviors, unrelated to the environment (e.g., “I read the newspaper”).

##### Treatment 2: social information

We implemented the social information treatment by providing information to subjects on the willingness to donate to the same environmental organization expressed by other users of the online platform. The pilot study was conducted before the main study and with different participants. Prior to Study 1, and with different participants, we conducted a pilot study on Prolific Academic, where we asked participants how much of their participation payment they would be willing to donate to WWF.^2^ Out of the 85 subjects recruited for the pilot, 72.9% claimed to be willing to make a donation if given a chance, with an average hypothetical donation amount of £0.2, corresponding to 40% of the participation payment. We provided this figure in the social information treatment, by telling subjects that “Last week, we conducted a similar survey on Prolific: participants were willing to donate on average 40% of their bonus to WWF UK.” This treatment draws from prior research on social information, showing how individuals tend to comply with behaviors that are perceived to be common among others from their same social environment (Goldstein et al., 2008).

Subjects in the *No social information* conditions did not receive any information while making the donation decision.

#### Measures

##### Manipulation check: environmental self-identity

Consistent with previous studies measuring environmental self-identity (Van der Werff et al., 2013a), we used three items: (a) “Acting environmentally friendly is an important part of who I am”; (b) “I am the type of person who acts environmentally friendly”; and (c) “I see myself as an environmentally friendly person” (Cronbach α = 0.91, *M* = 5.306, *SD* = 1.122). Respondents answered on a 7-point scale from “Completely disagree” to “Completely agree.” We construct an index of environmental self-identity by taking the unweighted average of the three questions.

##### Donation to an environmental organization

We measure pro-environmental behavior using an incentivized decision, namely donation of (any part of) the £1 bonus to WWF UK. We elicited the donation decision through an open question, so that participants could enter any amount between £0 and £1, with two decimals allowed. The beneficiary environmental organization was selected with the aim of maximizing its appeal to a wide audience: WWF UK is well known both in the United Kingdom and internationally, and is widely perceived as being politically neutral (Cracknell et al., 2013; Pharoah, 2017; Strauss, 2017).

##### Universalistic values

The literature on environmental self-identity models it as deriving from two main sources: past pro-environmental behaviors and values (Van der Werff et al., 2013b). We thus collected measures of universalistic values, in order to control for them in the empirical analysis. We used three survey questions from the European Social Survey (Davidov et al., 2008), asking respondents how much they felt similar to the individual described in different statements. The three statements we used to measure universalistic values are: (a) “It is important to this person that every person in the world is treated equally; everyone should have equal opportunities in life”; (b) “It is important to this person to listen to people who are different from him/her; even in case of disagreement, this person wants to understand them”; and (c) “This person strongly believes that people should care for nature. Looking after the environment is important to this person” (α = 0.62, *M* = 0.591, *SD* = 0.595). We construct an index of environmental values by taking the unweighted average of the three questions.

### Statistical Analysis

This section reports results of the analysis of the experimental data. We outline here the main steps we followed in the empirical analysis.

First, we investigate treatment effects on environmental self-identity. Namely, we use the data from the manipulation check on environmental self-identity to test whether the identity prime indeed had its intended effect.

We then study treatment effects on donation. We adopt different characterizations of the donation decision: first, we consider the overall average donation, including £0 donation amounts; second, we distinguish between the extensive margin, i.e., the decision of whether to donate or not, from the intensive margin, i.e., the choice of donation amount conditional on having donated. We use OLS regressions when the dependent variable is the donation amount and logit regressions when the outcome is an indicator equal to one if a positive donation is made.

In order to test whether the identity prime affects behavior through its influence on environmental self-identity, we follow the literature (Preacher and Hayes, 2008) and conduct mediated regression analysis. As recommended in the literature, we implement the mediation analysis through a total of 5,000 bootstrap samples, with 95% bias corrected and accelerated confidence intervals.

Finally, we examine a source of heterogeneity in treatment effects: prior pro-environmental behavior. It is likely that the impact of the prime depends on the number of environmental behaviors, asked about in the prime, that the individual actually performs. Since the rationale behind the prime relies on the assumption that claiming to perform regularly several pro-environmental behaviors will boost environmental self-identity, it is plausible that the prime will not affect the identity of individuals, who do not perform those behaviors frequently. We thus classify individuals depending on whether their reported engagement with the pro-environmental behaviors, listed in the prime, is above or below the median level of engagement in the sample. We define below median performers as the *Low frequency* group, and the above median ones as *High frequency* group and test whether the identity prime has a different impact on these two sets of subjects. Since engagement is not randomly allocated, but is likely to depend, among other things, on environmental values; and given that environmental values are also likely to independently influence the dependent variables, identity and donation, we control for them in the heterogeneity analysis.

### Results

#### Sample Characteristics

Overall, participants are aged between 18 and 73, 44% of them are female, 56% have university-level education and their average household income is between £2,000 and £3,000. Of the final sample, the identity priming only group comprises 95 participants (44 female); aged between 19 and 63; and 64% have completed a university-level qualification. The group exposed both to identity priming and social information comprises 104 participants (46 female); age ranges from 18 to 61; and 49% have completed a university-level qualification. The social information only group comprises 95 participants (39 female); aged between 18 and 58; with 59% of them having university-level qualification. Finally, the control group comprises 103 participants (48 female) with age ranging from 18 to 73; and 54% of them with university-level qualification. Supplementary Table S1, available in the online appendix, reports summary statistics and balance tests.

#### Impact of Identity Priming on Donation

**FIGURE 2:**
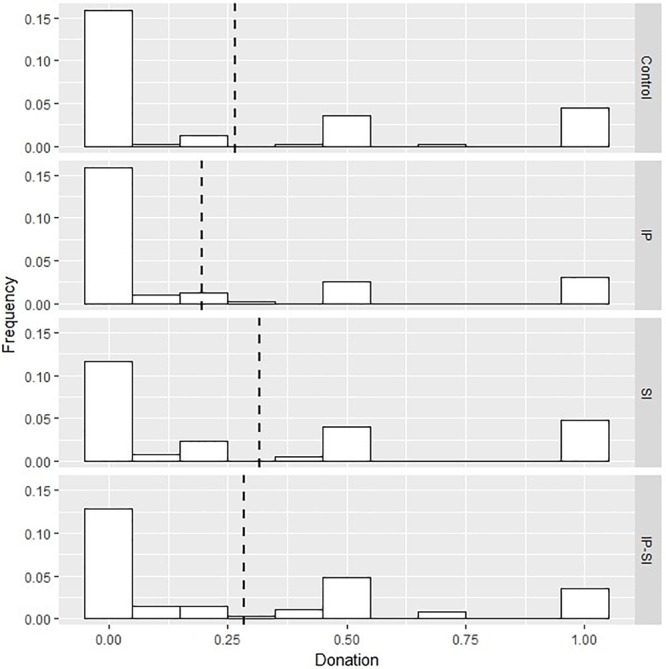
Distribution of donation per experimental condition, Study 1. IP, identity priming; SI, social information; IP – SI, identity priming and social information. Dashed line represents mean value.

Overall, subjects donated on average £0.27. This is in line with previous studies on contribution to charities, where participants donated around a third of their endowment (Bolton et al., 1998; Clot et al., 2016). Figure 2 reports the distribution of donation per experimental condition: across conditions, the distribution has a mode at £0, with smaller modes at £0.5 and £1.

Our identity priming is successful: participants reminded of their past pro-environmental behaviors exhibit a stronger environmental self-identity compared to the control group (Column 1, Table 2).^3^ Further, environmental self-identity is correlated to donation: the stronger environmental self-identity, the higher the average donation (*B* = 0.053, *p* < 0.01). This effect is due to the link between identity and donation on the extensive margin (*B* = 0.376, *p* < 0.01), while there is not a significant correlation on the intensive margin (*B* = 0.002, *p* > 0.10).

**Table 2:** Effect of identity priming and social information in Study 1.

	(1) Identity		(2) Average donation		(3) Extensive margin		(4) Intensive margin	
	*B*	*SE(B)*	*B*	*SE(B)*	*B*	*SE(B)*	*B*	*SE(B)*
IP	0.242**	0.112	–0.069	0.053	–0.264	0.296	–0.083	0.078
SI			0.051	0.053	0.477*	0.288	–0.053	0.070
IP^∗^SI			0.037	0.074	0.316	0.410	0.007	0.102
Const	5.186***	0.079	0.266***	0.036	–0.414**	0.201	0.668***	0.052
Obs	397		397		397		177	
*R*^2^	0.012		0.014				0.021	
Adj *R*^2^	0.009		0.006				0.004	
Log Likelihood					–267.651	
Akaike Inf. Crit.					543.3	
*F*	4.61^∗∗^		1.794				1.258	

*Linear regression (Columns 1, 2, and 4). Logit regression (Column 3). IP denotes the identity priming treatment, SI denotes the social information treatment. Standard errors reported in the SE(B) columns. ^∗^ significant at 10%; ^∗∗^ significant at 5%; ^∗∗∗^ significant at 1%.*

In spite of this positive correlation, the overall effect of the identity prime on donation is negative (Columns 2–4, Table 2), indicating no positive spillovers from prior pro-environmental behaviors to donation. Considering the effect of the prime on all participants exposed to the treatment, we find that, compared to the control group, the negative effect is significant only on the intensive margin (*B* = -0.112, *p* < 0.10). Similarly, Anderson Darling test reveals no differences between the distribution of average donation between participants in control and in identity only groups (Figure 2). To test whether the identity manipulation influenced average donation through its effect on environmental self-identity, we conduct mediation analysis.^4^ We detect partial and inconsistent mediation effects (MacKinnon et al., 2007): while average indirect effects are positive (*B* = 0.013, *p* < 0.10), the average direct effect is negative (*B* = -0.061, *p* < 0.10). These results suggest that reminding people of their past pro-environmental behaviors strengthens their self-identity, which, in turn, is positively related to donation. Nevertheless, for a given level of environmental self-identity, individuals donate less in the identity prime than in the control condition.

#### Heterogeneous Effects of Identity Priming

We now test whether the impact of the identity prime depends on the reported frequency of engagement in the environmental behaviors included in the manipulation. Consistent with the goal of the prime, these behaviors are indeed common among participants, with the median level of engagement across all behaviors equal to four (very frequently) on a five-point scale. Universalistic values predict whether a participant is classified in the *Low* or *High* group (*B* = 0.202, *p* < 0.01). They also predict environmental identity (*B* = 0.829, *p* < 0.01), as well as donation (*B* = 0.144, *p* < 0.01). Universalistic values, however, are not influenced by identity priming (*B* = 0.084, *p* > 0.10). Therefore, to have a clean effect of the number of behaviors recalled with respect to environmental values, we control for them in the regressions.

In order to explore heterogeneity of treatment effects by prior engagement with the behaviors, Table 3 shows separate regressions on self-identity and donation, among subjects in the *Low frequency* (Panel A) and in the *High frequency* (Panel B) groups. As hypothesized, the effect of the prime depends on reported frequency of engagement with the environmental behaviors. The impact on environmental self-identity increases in the number of behaviors: relative to the control group, only those in the *High frequency* group display higher self-identity (Panel B, Column 1), while negative but no significant effect is observed among *Low frequency* subjects (Panel A, Column 1). Both groups display lower donation levels compared to the control group, even though the negative impact is significant only in the *Low frequency* condition (Panel A, Columns 2–4).

**Table 3:** Effect of identity priming and social information for Low frequency **(A)** and High frequency **(B)** groups in Study 1.

	(1) Identity		(2) Average donation		(3) Extensive margin		(4) Intensive margin	
	*B*	*SE(B)*	*B*	*SE(B)*	*B*	*SE(B)*	*B*	*SE(B)*
**(A) Low frequency**		
IP	–0.194	0.126	–0.155**	0.121	–0.757*	0.814	–0.299**	0.118
SI			0.038	0.064	0.427	0.437	–0.058	0.068
IP^∗^SI			0.078	0.087	0.534	0.194	0.212	0.143
Univ	0.745***	0.085	0.132***	0.029	0.774***	0.300	0.108**	0.047
Const	2.193***	0.079	–0.255	0.121	–3.517***		0.215	0.203
Obs	292		292		292		122	
*R*^2^	0.225		0.105				0.101	
Adj *R*^2^	0.219		0.092				0.071	
Log Likelihood					–184.152	
Akaike Inf. Crit.					378.304	
*F*	41.912^∗∗^		8.395^∗∗∗^				3.304^∗∗^	
**(B) High frequency**		
IP	0.507***		–0.027	0.065	–0.103	0.353	–0.022	0.087
SI			0.037	0.054	0.428	0.296	–0.057	0.070
IP^∗^SI			0.023	0.023	0.357	0.502	–0.047	0.114
Univ	0.769***		0.138***	0.035	0.743***	0.202	0.079	0.057
Const	2.095***		–0.281*	0.143	–3.939***	0.845	0.336	0.215
Obs	303		303		303		145	
*R*^2^	0.299		0.056				0.032	
Adj *R*^2^	0.294		0.044				0.005	
Log Likelihood					–198.261	
Akaike Inf. Crit.					406.521	
*F*	63.990^∗∗∗^		4.447^∗∗∗^				1.169	

*Linear regression (Columns 1, 2, and 4). Logit regression (Column 3). IP denotes the identity priming treatment, SI denotes the social information treatment, Univ denotes universalistic values. Standard errors reported in the SE(B) columns. ^∗^ significant at 10%; ^∗∗^ significant at 5%; ^∗∗∗^ significant at 1%.*

Additional analysis, reported in Table 2 of the online Supplementary Material, confirms the statistical significance of the heterogeneity results. Namely, we pool the entire sample and regress experimental outcomes on the identity priming dummy and its interaction with an indicator for *High Frequency* subjects. The coefficient on the interaction term is statistically significant in the regressions featuring environmental identity, average donation and the probability to donate as dependent variables.

#### Impact of Integration of Identity Priming and Social Information on Donation

Consistent with previous studies, the social information treatment has a positive impact on donation: exposing participants to others’ moral behavior results in higher average donation (Column 2, Table 2). Distinguishing between the extensive and intensive margin, we see that social information positively and significantly affects the probability to make a positive donation (Column 3, Table 2), and negatively the amount donated conditional on making a positive donation (Column 4, Table 2). Moreover, the positive sign of the interaction term between the self-identity and social information treatments indicates that social information offsets the negative impact on donation of the self-identity prime; the lack of significance shows that the combined effect is roughly consistent with an additive effect of the two stimuli. This additive effect results in significant higher regression coefficient for the joint identity-social treatment compared to the identity only condition, both for average donation (*p* < 0.10) and for likelihood to donate (*p* < 0.01). Additionally, Anderson Darling test shows that the two samples belong to different distributions (*p* < 0.01) (Figure 2).

### Discussion

In Study 1, we show that reminding individuals of their past pro-environmental behaviors results in higher reported environmental self-identity, and that higher self-identity is positively correlated to pro-environmental action within the experiment. However, we also show that the overall impact of priming self-identity on subsequent behavior is negative, although not significantly so. Mediation analysis reveals the mechanism underlying this effect: whereas identity priming has an indirect positive effect on donation through environmental self-identity, it also directly negatively affects donation. The negative coefficient on the priming treatment indicator indicates that no positive spillovers from past to future environmental behaviors occur within our experiment, and are at prima facie suggestive of the presence of moral licensing (Khan and Dhar, 2006; Sachdeva et al., 2009): remembering past pro-environmental behaviors provides participants with moral credits, which legitimate them to contribute less in the subsequent environmental decision.

Heterogeneity analysis, however, tells a different story. The identity prime does not have the same effect on all subjects. Namely, it increases environmental self-identity, relative to the control, only among subjects who engage in the behaviors contained in the prime on a recurring basis. If moral licensing were at work, we would expect negative spillovers from the prime to be most pronounced among highly engaged participants. On the contrary, it is unengaged subjects, who experience a decrease in self-identity as a result of the prime, who drive the negative overall impact of identity priming on donation. It is important to highlight, however, that, even among highly engaged subjects, the identity priming does not lead to positive spillovers: donation levels among the most engaged participants are still lower than those of control group subjects.

We identify a way to mitigate the negative spillovers from the identity prime, i.e., social information. Making others’ moral behavior salient encourages individuals to act in a norm-consistent way. Negative spillovers from the prime are completely offset by social information: when the two treatments are combined, average donation is not significantly different from that in the control group. Two alternative psychological mechanisms may explain this result. On the one hand, the effect of social information may be driven by the threat to one’s moral self, coming from not complying with others’ moral behavior. On the other hand, contribution ethic would explain why the perception of having done one’s share, fostered by the identity prime, is offset by the realization that others have also contributed to the common good.

In order to disentangle the effect of these two psychological mechanisms, and to provide clear explanation of why social information neutralizes negative spillover, in Study 2 we further investigate how past pro-environmental deeds affect subsequent behaviors depending on the prevalent conduct in a reference group. We argue that, if contribution ethic is the main driver of the behavior we observe in Study 1, then the identity prime will lead to a stronger anchoring between own and others’ pro-environmental decisions. The causal link goes as follows: reminding individuals of their past pro-environmental behaviors makes them feel more strongly to have already contributed enough to the common good; this feeling then makes them anchor their subsequent behavior more to the social norm. Namely, for others’ low levels of contribution, they feel justified to make smaller contributions than in the absence of the prime; while for others’ high levels of contribution, the realization that others are also doing their share neutralizes the negative spillover. Therefore, we expect the identity prime to increase the share of individuals behaving as conditional cooperators, if contribution ethic is at work.

We designed Study 2 to collect further evidence on the sign of spillover effects, to identify the mechanism behind them and to rule out alternative explanations for their occurrence. First, we replicate the identity prime treatment of Study 1. Second, to test whether contribution ethics can explain the combined effect of identity priming and social information, we elicit donation decisions both in terms of unconditional donation, and of donation conditional on other subjects’ donation level. This allows us to investigate treatment effects on the full donation profile and to investigate whether the identity prime fosters conditional cooperation. Third, as we did not observe positive spillover even among highly engaged subjects, we try to investigate whether the lack of spillovers is due to the weak link, generated by the identity prime, between past deeds and one’s moral self. We do so by augmenting the identity prime with a goal commitment exercise, another common behavioral policy. As previous experiments, we implement goal commitment with an attribution recall task (Mukhopadhyay et al., 2008). We believe attribution recall to be an effective strategy to increase the connection between simple past pro-environmental behaviors and one’s moral self, because it requires subjects to make the moral drivers behind their past behaviors explicit.

In sum, Study 2 extends Study 1 in two ways. First, we test whether goal commitment is an effective strategy to promote positive spillover effects. Second, we elicit participants’ donation decisions as unconditional and conditional to others’ donation.

## Study 2

### Materials and Methods

#### Participants and Procedure

We conducted Study 2 on the same online platform, and using the same payment scheme, as Study 1, but with a different sample. In total, 471 Prolific Academic users completed the experiment. Participants were randomly assigned to the treatments in a between subjects design. The experimental protocol differs from that of Study 1 under three respects. First, since the goal commitment treatment builds on the identity prime one, and can therefore be administered only to subjects who received the identity prime, Study 2 features 3 experimental conditions: control, identity prime only and identity prime plus goal commitment. Second, we elicit the donation decision both as unconditional donation amount (unconditional donation), and as a profile of donation amounts, conditional on all the possible levels of average donation by the other participants in the experiment (conditional donation). Third, since the instructions for the donation task are longer than in Study 1, the post-donation survey begins with the questions on environmental values. The Supplementary Material available online reports the entire text of Study 2 instructions.

#### Materials

##### Treatment 1: identity priming

Identity priming takes place in the same way as in Study 1. Table 4 shows that, consistent with Study 1, the eight pro-environmental behaviors are common among the Study 2 sample.

**Table 4:** Actions included in the environmental priming exercise and frequency of reported engagement, Study 2.

Action	*M*	*SD*
I turn off the lights when no one is in the room	4.271	0.791
I do not throw litter on the street	4.526	1.029
I recycle newspapers, glass, aluminum, motor oil, or other items	3.942	1.096
I turn off electrical appliances (to save energy)	3.842	1.038
I move around by bike and/or public transportation	2.977	1.406
I buy a less polluting product if there is a choice in the shop	2.974	1.114
I use reusable shopping bags at grocery stores instead of the standard plastic or paper bags	3.878	1.230
I leave a clean spot after a picnic	4.700	0.708
Total	3.877	0.551
Number observations: identity priming and identity priming plus goal commitment	310	

##### Treatment 2: goal commitment

Drawing from previous research (Mukhopadhyay et al., 2008), we activate participants’ focus on goal commitment by asking them to recall and list three reasons why they performed the pro-environmental behaviors reported in identity priming. We framed the task in the form of an open-ended question, and participants were provided with three boxes to write the attributions.

#### Measures

##### Manipulation check: environmental self-identity

We measure environmental self-identity with the same items as in Study 1. Items form a reliable scale (Cronbach α = 0.91, *M* = 5.317, *SD* = 1.100).

##### Environmental attributions recalled

We classify the reasons, listed by subjects in the goal commitment exercise, as driven by environmental motives or not, and count the number of environmental attributions mentioned by each participant. This variable ranges between 0 and 3.

##### Universalistic values

Universalistic values are measured with the same items as in Study 1. Items form a consistent scale (Cronbach α = 0.62, *M* = 0.605, *SD* = 0.578).

##### Donation to an environmental organization

As in Study 1, we asked respondents whether they wanted to donate any part of the additional bonus of £1 to WWF UK. In addition to this, we used the strategy method to elicit donation amounts as a function of other subjects’ average donation. We implemented the strategy method as follows: after entering the unconditional donation, subjects filled a “contribution table,” where they had to indicate, for each of the 11 possible amounts donated on average by other participants (in £0.1 increments), how much they were willing to donate to WWF UK (Fischbacher et al., 2001). We randomized whether others’ donation was displayed in increasing or decreasing order to prevent anchoring effects. To ensure incentive compatibility and provide a motivation to take both the unconditional and conditional decisions seriously, we told participants that each of them had the same probability to be drawn as the payoff relevant one at the end of the experiment. This means that half of the participants paid according to their unconditional donation. The average donation amount by this group of subjects determined the payoff of subjects paid according to their conditional donation amount.

### Statistical Analysis

We adopt the same empirical strategy as in Study 1 to investigate treatment effects on identity and unconditional donation. To this, we add the analysis of treatment effects on conditional donation. We classify subjects’ donation profiles according to the types identified by the literature on conditional cooperation (Fischbacher et al., 2001). To this end, as in Fischbacher et al. (2001), we compute Spearman’s rank correlation coefficient between own and other’s donation: among coefficients significant at the 5% level, we classify positive ones as identifying conditional cooperators and negative ones as denoting anti-cooperators. Subjects, whose conditional donations are not influenced by others’ choices, are classified as unconditional contributors. We use visual inspection to classify hump-shaped conditional donation profiles, and to assign the remaining profiles to the existing categories, whenever possible. To take into account the multiple observations generated by the strategy method for each subject, when investigating treatment effects on conditional donation, we run a mixed model with random effects at individual level.^5^

### Results

#### Sample Characteristics

Participants in Study 2 are aged between 18 and 79 years old, 52% are female, 58% have university-level education, and their average household income is between £3,000 and £5,000. Of the final sample of 471^6^, the identity priming group comprises 156 participants (77 female); age ranges from 19 to 79; and 61% has completed a university-level qualification. The group exposed both to identity priming and social information comprises 154 participants (83 female); age ranges from 18 to 69; and 55% has completed a university-level qualification. The control group comprises 161 participants (89 female); age ranges from 18 to 63; 59% has completed a university-level qualification. The average household income for all groups is between £3,000 and £5,000. Table 2, available in the online Supplementary Material, reports summary statistics and balance test for the Study 2 sample.

#### Impact of Identity Priming on Donation

**FIGURE 3:**
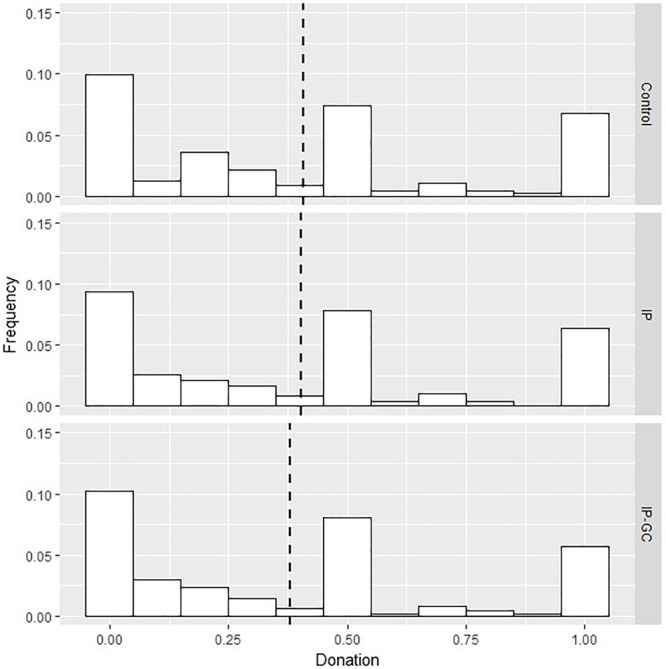
Distribution of donation per experimental condition, Study 2. IP, identity priming; IP – GC, identity priming and goal commitment. Dashed lines represent mean values.

The overall average unconditional donation is £0.4 out of £1, slightly higher compared to Study 1 (*t*-test, *p* < 0.01) and to previous studies (Bolton et al., 1998; Clot et al., 2016). Relative to Study 1, the distribution of donation in all three treatments displays a less pronounced mode at 0, and larger shares of subjects contributing half and all of the bonus (Figure 3). We can only speculate on the possible causes of the difference between Study 1 and Study 2 donation patterns, since the two studies were conducted months apart, with different samples and using slightly different protocols. One reason for the difference may lie in the higher household income level of Study 2 participants (*t*-test, *p* < 0.01), as previous studies found a positive relationship between income and charity support (Lee and Chang, 2007), as well as between income and pro-environmental behaviors (Clark et al., 2003).

The results on the identity prime in Study 2 are broadly consistent with those from Study 1. First, environmental self-identity is significantly higher among subjects exposed to identity priming (Column 1, Table 5).^7^ Second, participants who report higher environmental self-identity donate more (*B* = 0.070, *p* < 0.01), both on the extensive (*B* = 0.357, *p* < 0.01) and on the intensive margins (*B* = 0.053, *p* < 0.01). Third, the effect of the identity prime on unconditional donation is always not statistically significant, and generally negative (Columns 2–4, Table 5). Also, when comparing donation levels of all the participants exposed to identity priming with the control group, we find no difference for any formulation of the dependent variable. In addition, the distribution of average donation in identity priming only does not differ with the one of the control group (Anderson Darling test, *p* > 0.10). We can thus exclude that the prime had a positive or negative effect on unconditional donation: no positive spillovers nor moral licensing appear to occur within Study 2. Results of mediation analysis are also consistent with Study 1: average indirect effects are positive and significant (*B* = 0.027, *p* < 0.01), while the average direct effect is negative but not significant (*B* = -0.043, *p* > 0.10). Even if this pattern is qualitatively in line with Study 1, in Study 2 the average direct effect is weaker. Hence, also in Study 2 our results suggest that reminding individuals of their past pro-environmental behaviors strengthens their environmental self-identity, which, in turn, is positively related to donation. Nonetheless, even in the presence of a weaker negative direct effect, the positive indirect effect of priming identity does not induce an increase in subsequent pro-environmental behavior.

**Table 5:** Effect of the self-identity prime and goal commitment in Study 2.

	(1) Identity		(2) Average donation		(3) Extensive margin		(4) Intensive margin	
	*B*	*SE(B)*	*B*	*SE(B)*	*B*	*SE(B)*	*B*	*SE(B)*
IP	0.375***	0.123	–0.004	0.041	0.062	0.803	–0.015	0.042
IP - GC		0.122	–0.028	0.041	–0.045	0.250	–0.033	0.042
Const	5.070***	0.086	0.407***	0.029	0.886***	0.173	0.575***	0.029
Obs	471		471		471		334	
*R*^2^	0.026		0.002				0.002	
Adj *R*^2^	0.024		–0.004				–0.004	
Log Likelihood					–283.887			
Akaike Inf. Crit.					573.77			
*F*	12.59^∗∗∗^		1.794				0.311	

*Linear regression (Columns 1, 2, and 4). Logit regression (Column 3). IP denotes the identity priming treatment, GC stands for goal commitment. Standard errors reported in the SE(B) columns. ^∗^ significant at 10%; ^∗∗^ significant at 5%; ^∗∗∗^ significant at 1%.*

#### Heterogeneous Effects of Identity Priming

We now turn to the study of heterogeneous treatment effects on the basis of subjects’ reported level of engagement with the behaviors listed in the identity prime. As in Study 1, the median subject reported to perform 4 out of 5 behaviors. Again, universalistic values act as potential confounder by predicting whether a participant is in the *Low* or *High frequency* group (*B* = 0.139, *p* < 0.05), and the dependent variables: environmental identity (*B* = 0.891, *p* < 0.01), as well as donation (*B* = 0.173, *p* < 0.01). Universalistic values are balanced among the experimental conditions (*B* = 0.029, *p* > 0.10), so that they can be used as control.

Table 6 reports regressions on the dependent variables, distinguishing between the *Low frequency* (Panel A) and the *High frequency* (Panel B) groups. In line with Study 1, participants’ reaction to the identity manipulation differs depending on prior engagement: compared to the control group, environmental self-identity is higher only among subjects in the *High frequency* group (Panel B, Column 1). As for donation, even not significantly, the *Low* and the *High frequency* groups show opposite effects: compared to the control group, below-median participants display lower, whilst above-median higher donation (Columns 2–4).

**Table 6:** Effect of identity priming and goal commitment for *Low frequency* **(A)** and *High frequency* **(B)** groups in Study 2.

	(1) Identity		(2) Average donation		(3) Extensive margin		(4) Intensive margin	
	*B*	*SE(B)*	*B*	*SE(B)*	*B*	*SE(B)*	*B*	*SE(B)*
**(A) Low frequency**		
IP	0.129	0.107	–0.039	0.047	–0.196	0.309	–0.022	0.051
IP-GC			–0.091*	0.047	–0.230	0.308	–0.074	0.148
Univ	0.827***	0.081	0.189***	0.0029	0.940***	0.202	0.146***	0.034
Const	1.745***	0.334	–0.352***	0.118	–2.813***	0.805	–0.038	0.147
Obs	312		312		312		211	
*R*^2^	0.252		0.139				0.101	
Adj *R*^2^	0.247		0.131				0.088	
Log Likelihood					–183.673			
Akaike Inf. Crit.					375.347			
*F*	51.205^∗∗∗^		16.576^∗∗∗^				7.785^∗∗∗^	
**Panel (B): High frequency**		
IP	0.550***	0.103	0.019	0.049	0.246	0.346	0.000	0.049
IP-GC			0.029	0.049	0.100	0.329	0.027	0.049
Univ	0.895***	0.075	0.166***	0.029	1.062***	0.205	0.086**	0.034
Const	1.474***	0.311	–0.260**	0.122	–3.287***	0.815	0.213	0.146
Obs	320		320		320		237	
*R*^2^	0.375		0.096				0.029	
Adj *R*^2^	0.371		0.088				0.016	
Log Likelihood					–166.939			
Akaike Inf. Crit.					341.878			
*F*	95.06^∗∗∗^		11.238^∗∗∗^				2.284^∗^	

*Linear regression (Columns 1, 2, and 4). Logit regression (Column 3). IP denotes the identity priming treatment, GC denotes the goal commitment treatment, Univ denotes universalistic values. Standard errors reported in the SE(B) columns. ^∗^ significant at 10%; ^∗∗^ significant at 5%; ^∗∗∗^ significant at 1%.*

These results are confirmed when we investigate heterogeneous treatment effects on the full sample. Table 4 of the online Supplementary Material shows a statistically significant and positive interaction term between our prime and the dummy *High Frequency* only when predicting environmental self-identity, but not for donation.

#### Impact of Integration of Goal Commitment and Identity Priming on Donation

In contrast with our hypothesis, augmenting the identity prime with the goal commitment exercise does not affect the sign, magnitude or statistical significance of the spillover effects of past pro-environmental behavior: participants exposed to both treatments donate less, even if not significantly so, than those in the identity prime only group (Columns 2–4, Table 5). Similarly, no difference is observed between the distribution of donation in the identity prime only and when combined with goal commitment (Anderson-Darling test: *p* > 0.10) (Figure 3).

We exploit data from the goal commitment exercise to unpack further this result. Participants in the *Low frequency* group listed fewer environmental reasons for performing the behaviors included in the prime during goal commitment, relative to subjects in the *High frequency* group (*B* = -0.403, *p* < 0.01). They also donated significantly less than participants in the control group, and marginally less than individuals, with a similar engagement level, exposed to the identity prime only (Panel A, Columns 2–4, Table 6).

#### Impact of Identity Priming and Goal Commitment on Conditional Donation

**FIGURE 4:**
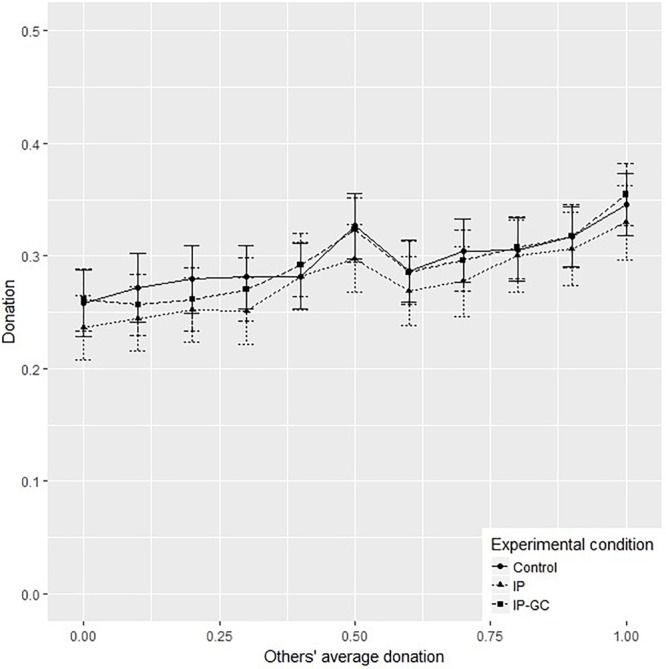
Mean of conditional donation by treatment, Study 2. IP, identity priming; IP – GC, identity priming and goal commitment. Error bars represent 95% confidence intervals.

We conclude the empirical analysis of Study 2 by reporting results on conditional donation. Figure 4 displays donation levels, conditional on others’ donation, by experimental treatment. Consistent with other studies, participants are willing to give more as others’ average donation level increases (Fischbacher et al., 2001; Fischbacher and Gachter, 2010; Préget et al., 2016). This pattern is in line with the results on the social information treatment in Study 1, and is confirmed by regression analysis. Regressing subjects’ own donation decision, elicited with the strategy method, on the level of others’ average donation, we find that the former significantly increases with the latter (Table 7).

**Table 7:** Effect of identity priming, goal commitment and level of others’ donation on conditional donation, Study 2.

	Conditional donation	
	*B*	*SE(B)*
IP	–0.019	0.037
IP-GC	–0.003	0.037
Other’s donation	0.077***	0.007
Const	0.258	0.026
Obs	5181	
No. clusters	471	
Log Likelihood	1296.991	
Akaike Inf. Crit.	–2581.983	

*IP denotes the identity priming treatment, GC denotes the goal commitment treatment. Standard errors reported in the SE(B) columns. ^∗^ significant at 10%; ^∗∗^ significant at 5%; ^∗∗∗^ significant at 1%.*

The set of types, which we derive from the classification of participants’ donation profiles, is also consistent with those identified in the literature on public good games. We classify 53.7% of subjects as unconditional cooperators, of which 33.1% are free-riders; 21% as conditional cooperators; 5.5% as anti-cooperators; and 2.5% with hump-shaped donation profiles. We could not classify 17.2% of participants according to these types. These shares differ from those observed in the existing literature on public good games (Fischbacher et al., 2001; Fischbacher and Gachter, 2010; Préget et al., 2016). Many factors may explain this discrepancy, among which the difference in the experimental decision is likely to play a role.

We next explore whether the share of different donation profiles is affected by the treatments. Table 8 shows that subjects in the identity prime treatment are significantly more likely to be classified as conditional cooperators (*t*-test, *p* < 0.05). This increase mirrors a decrease of similar magnitude in the share of unconditional donors in the identity priming condition (*t*-test, *p* < 0.10).

**Table 8:** Conditional donation profiles by treatment, Study 2.

Donation profile	Control	IP
Unconditional cooperator:	0.705^a^	0.618^a^
Free rider (donation < 0.3)	0.432	0.382
Medium (0.3 ≤ donation < 0.7)	0.115	0.084
High (donation ≥ 0.7)	0.158	0.151
Conditional cooperator	0.194^1^	0.287^1^
Anti-cooperator	0.065	0.068
Hump-shaped	0.036	0.028
Total classified	139	251

*IP denotes the identity priming treatment. Letters and numbers represent the significance of pairwise comparisons per experimental condition with T-test with different variances, two tails. Same letter in the row represents a significant difference with p < 0.10; same number p < 0.05*

Finally, we conduct regression analysis also on the conditional donation decisions. We obtain similar results as when we analyze treatment effects on unconditional donation: the impact of the identity prime is negative but not significant for any formulation of the dependent variable (Table 7).

### Discussion

The results from Study 2 overall support those of Study 1. Reminding individuals of their past pro-environmental behaviors leads to significantly higher reported environmental self-identity, which is, in turn, related to higher donation. However, overall, the impact of our manipulation on following pro-environmental decisions is negative, albeit not statistically significant. Once again, our results illustrate that making salient participants’ morality does not lead to positive spillovers.

Even though we qualitatively replicate all the Study 1 results in Study 2, the overall significance level of our estimates is lower in the second experiment. We suggest potential explanations for this. First, the sample in Study 2 features, on average, wealthier participants. Higher income may be associated with higher donations and with lower sensitivity to the small incentives provided within the experiment (Clark et al., 2003; Lee and Chang, 2007; Andreoni et al., 2017). Indeed, we observe higher giving in Study 2, both relative to Study 1 and to previous experiments (Bolton et al., 1998; Clot et al., 2016). Second, the elicitation of the donation decision differs between the two studies. Asking participants to make two choices and randomly selecting the payoff-relevant one may induce them to take each decision less seriously and translate in noisier decision outcomes. Third, the use of the strategy method may affect elicited donations. Evidence on the effect of the elicitation method on experimental subjects’ behavior is mixed: whereas some studies observe consistent results across direct and strategy methods, others detect weaker treatment effects when the dependent variable is elicited through indirect strategies compared to direct ones (see Brandts and Charness, 2011 for a review).

As in Study 1, we observe strong heterogeneous effects of the identity prime, depending on subjects’ engagement with the behaviors listed in the prime. The identity prime is positively associated with significantly higher levels of reported self-identity only among individuals who often engage in the prime’s pro-environmental behaviors, but has no impact on subsequent donation among them. On the other hand, there is a negligible impact of the priming on identity and on donation among participants who seldom perform the behaviors.

Our results on conditional donation are consistent with the positive impact of social information that we observed in Study 1. Social information, it is argued, influences behaviors toward the desired social outcome thanks to the underlying positive correlation between one’s and others’ moral behavior (Schultz, 1999; Schultz et al., 2007; Goldstein et al., 2008; Nolan et al., 2008; Allcott, 2011; Ferraro et al., 2011; Nomura et al., 2011; Toelch et al., 2011; Harries et al., 2013). We confirm this mechanism thanks to the use of the strategy method. Further, the higher presence of conditional cooperators in the identity priming condition suggests that the lack of positive spillovers from the identity priming is primarily caused by contribution ethic, as individuals tend to be more compliant with the prevalent behavior.

Finally, contrary to our expectation and to prior evidence (Fishbach and Dhar, 2005; Fishbach et al., 2006; Mukhopadhyay et al., 2008), goal commitment does not reverse the sign of spillovers from the identity priming exercise. On the contrary, we illustrate that goal commitment may even intensify the negative spillovers caused by the identity prime. Heterogeneity analysis reveals that the significant negative effect of goal commitment primarily arises among those subjects who report low engagement levels in the identity manipulation –the same participants who recalled fewer environmental reasons in the goal commitment exercise.

## General Discussion

We have attempted to experimentally test how past pro-environmental behaviors affect subsequent environmental decisions. In two online, incentive-compatible studies, we randomly manipulate environmental self-identity, by reminding participants of their past pro-environmental behaviors, and then ask them to make a pro-environmental decision. This set-up allows us to study the sign and magnitude of spillovers generated by the identity prime. Further, we explore the heterogeneous impact of the prime on the basis of individuals’ responses to it. Finally, we investigate whether common behavioral policies, when integrated with identity priming, can affect the sign and magnitude of spillovers. Specifically, Study 1 focuses on social information, whereas Study 2 on goal commitment.

### Sign and Magnitude of Spillover Effects

In both studies, identity priming does not result in positive spillover. Even when environmental self-identity is boosted by reminding individuals of a set of environmentally friendly behaviors they performed in the past, this positive effect on environmental self-identity hardly translates into higher levels of subsequent pro-environmental decisions. Rather, when asked to renounce to part of their participation endowment in support of an environmental organization, treated participants end up contributing lower amounts. This finding is at prima facie consistent with a moral credit model (Sachdeva et al., 2009), which posits that the heightened sense of morality, resulting from previous moral actions, justifies reduced moral behaviors in subsequent choices. Our heterogeneity analysis, however, tells a more nuanced story.

A puzzling aspect of our results is that our identity prime replicates a methodology that previous studies find effective in inducing positive spillovers (Cornelissen et al., 2008; Van der Werff et al., 2013a, 2014a,b). We speculate that the sign of spillovers depends on how the psychological costs of behavioral inconsistency compares with the inherent costs of behaving morally. Indeed, previous studies achieving consistency mainly relied on self-reported measures (Cornelissen et al., 2008; Van der Werff et al., 2013a, 2014a,b) or effortless behaviors (Cornelissen et al., 2008; Van der Werff et al., 2014b). Our behavioral outcome instead involves higher inherent personal cost. Hence, the negative spillovers we detect may conceivably be explained by the different relative weights of inconsistency with one’s moral self and cost to behave morally faced by subjects in our studies. While we cannot formally test this, we support this speculation by noting that our results are consistent with evidence of moral licensing in the domain of charitable contribution (Khan and Dhar, 2006; Sachdeva et al., 2009; Clot et al., 2016), a very similar setting to ours.

Further research is needed to systematically investigate how the nature, and particularly the cost, of the dependent variable affects the sign and magnitude of spillovers from this type of intervention. The interaction between the features of past and subsequent moral environmental behaviors is also likely to matter. Indeed, our results are consistent with different theoretical perspectives. For instance, cognitive dissonance theory (Festinger, 1962) claims that the higher (lower) the similarity among two behaviors, the higher (lower) the costs associated with behavioral inconsistency, and the higher (lower) the likelihood that one will engage in both (Thøgersen, 2004; Thøgersen and Crompton, 2009). Thus, lack of consistency in our experiment may also derive from the difference between the outcome variable, donation to an environmental charity, and the behaviors included in the identity prime, rather than from the cost of the donation decision, as we hypothesize. Testing between different theories will require experimental studies, varying systematically the nature of both prior and subsequent behaviors.

### Heterogeneity of Spillover Effects

Our heterogeneity analysis also supports this view. The fact that negative spillovers are more pronounced among subjects with low levels of engagement in the behaviors included in the prime is consistent with the literature in two ways. First, according to the self-perception theory (Bem, 1972), lack of engagement results in a negative or non-significant inference of attitude from past deeds, as observed in our studies and in previous research (Cornelissen et al., 2008; Van der Werff et al., 2013a, 2014b). Second, if past pro-environmental behaviors are too weakly connected to the moral self, or if they are not motivated by environmental considerations, they will not prompt cross-behavioral consistency. In a similar vein, it is possible to interpret the behavior of highly engaged participants, for whom we observe no positive spillovers either, in spite of the positive effect of the prime on their environmental self-identity. While they infer from the prime that they are environmentally friendly individuals, the signaling power of our manipulation is conceivably too low to give rise to positive spillovers for these individuals, given how common the target behaviors are (Thøgersen and Crompton, 2009).

### Policy Implications

Our studies investigate the interplay of multiple nudges when implemented in conjunction. First, we show how a behavioral tool, social information, successfully mitigates the negative spillovers caused by identity priming (Study 1). We argue that the negative spillovers from the identity priming may result either from a heightened sense of morality (Sachdeva et al., 2009) or from the feeling of having already done one’s own “fair share” (Kahneman et al., 1993; Guagnano et al., 1994; Thøgersen and Crompton, 2009). Social information may offset the former effect by inducing a feeling of moral incompleteness; and may overcome the latter by alleviating the feeling of unequal participation to the common cause. In Study 2, we provide evidence in favor of the second mechanism: the larger share of conditional cooperators in the identity priming treatments suggests that the intensity of negative spillovers depends on the prevailing norm. This implies that social influence mitigates such spillovers mainly because it corrects subjects’ misperception that they contribute more than others.

Second, we find that goal commitment, which is found in other context to offset moral licensing (Fishbach and Dhar, 2005; Fishbach et al., 2006; Mukhopadhyay et al., 2008), has no effect when combined with identity manipulation (Study 2). This result was the opposite of what we expected, since the goal commitment exercise, by making more salient the reasons for prior behaviors, was meant to strengthen the connection between those behaviors and the moral self and increase the psychological cost of inconsistency. The negative interplay between identity priming and goal commitment is conceivably due to the fact that those who fail to engage in prior environmental behaviors, also can recall few environmental motives behind those behaviors. Thus, for them, the combination of the two nudges undermined even more their self-identity, resulting in lower level of donation. This finding is in line with previous studies suggesting that, whenever it is possible to attribute the same behavior to different reasons, positive cross-behavioral spillovers are hardly achieved (Cornelissen et al., 2008).

Another possible reason why our results do not replicate those of other studies using similar methods may lie in the specific nature of environmental decision. While the environment is commonly considered part of the moral sphere (Thøgersen, 1996; Klöckner, 2013), it is in our opinion likely to fall within the category of imperfect duties, in spite of the severity of climate change. Fulfilling imperfect duties has a positive impact of one’s moral self, but not following them does not threatened one’s morality (Wiltermuth et al., 2010; Kant, 2013). Reminding individuals that they do not comply with imperfect duties does not activate compensating decisions, as it is usually observed in other moral decision settings (Sachdeva et al., 2009; Zhong et al., 2010; Jordan et al., 2011), but rather results in negative consistency or no effect (Cornelissen et al., 2008; Van der Werff et al., 2013a, 2014a,b). This reasoning would suggest caution when extending the literature about moral behaviors to the environmental domain. A formal test of this argument would require an investigation into individual perception of environmental behaviors relative to other moral decisions.

Our results have important theoretical and practical implications. First, they contribute to the literature on spillover effects, by experimentally testing the impact of priming past environmental behaviors on subsequent decisions, through an incentive-compatible design and with a large and heterogeneous sample. Second, our heterogeneity analysis highlights the importance of targeting identity priming interventions to minimize negative spillovers. Finally, we identify nudges that can offset or exacerbate the negative spillovers from identity priming. In a world characterized by increasing exposure to behavioral policies, practitioners should pay attention to the unintended consequences of combining multiple behavioral tools.

Our work also presents some limitations. First, the significance of our results is rather weak, especially in Study 2. Even though we provide plausible explanations, future research should test the robustness of our results. Second, our results do not shed light on the process through which, according to our interpretation of the empirical results, individuals balance the costs of behavioral consistency against the those of acting morally. Finally, it is certainly disappointing that we do not succeed in generating positive spillovers, even among the most engaged subjects.

## Conclusion

To summarize, we study how past pro-environmental behaviors affect subsequent environmental decisions, and the role played by common behavioral policies. Overall, the two experimental studies – carried out on a heterogeneous sample and in an incentive compatible way – provide evidence that past pro-environmental actions strengthen environmental self-identity, but, at the same time, fail to promote following pro-environmental decisions. Even worse, they generate negative spillovers among subjects who engage less in pro-environmental behaviors. Finally, we show that, depending on which behavioral strategy is put in place, negative spillovers can be either mitigated or magnified.

## Ethics Statement

The wider project of which this study was part (COBHAM) underwent ethical review, and received the approval of Politecnico di Milano’s Ethical Committee. All participants gave written informed consent in accordance with the Declaration of Helsinki.

## Author Contributions

VF conceived the study, led the analysis of data, and the writing of the paper. Gd’A contributed to the conception and the design of the study, to the preparation of experimental material, and to the writing the manuscript. MT contributed to the conception and the design of the study, to the writing of the manuscript, and was in charge of overall direction and planning.

## Conflict of Interest Statement

The authors declare that the research was conducted in the absence of any commercial or financial relationships that could be construed as a potential conflict of interest.

## Footnotes

1 Despite their relatively recent adoption as data collection tools, online labor platforms offer reliable results, as shown by replications of well-known experiments (Paolacci et al., 2010; Horton et al., 2011; Suri and Watts, 2011; Crump et al., 2013).

2 The Supplementary Material available online reports the entire text of the pilot study.

3 We pool the self-identity only and self-identity plus social information treatments in Column 1 because the prime and manipulation check preceded the social information treatment. Indeed, the impact of the identity prime on self-identity does not differ between the two treatments. Result available upon request.

4 The independent variable does not need to predict the dependent variable to test mediation effects (Shrout and Bolger, 2002; James et al., 2006).

5 Hausman test confirmed that random effects are appropriate in our setting.

6 We dropped 23 participants from the sample: 22 of them because of missing data, and 1 because she repeated the task twice.

7 As in Study 1, we pool the self-identity only and self-identity plus goal commitment treatments in Column 1. Even if the manipulation check followed goal commitment, the impact of the identity prime on self-identity does not differ between the two treatments. Result available upon request.

## References

[B1] Allcott H. (2011). Social norms and energy conservation. *J. Public Econ.* 95 1082–1095. 10.1016/j.jpubeco.2011.03.003

[B2] Andreoni J. Nikiforakis N. Stoop J. (2017). “Are the rich more selfish than the poor, or do they just have more money? A natural field experiment,” Discussion paper. *Natl. Bur. Econ. Res.* 10.3386/w23229

[B3] Ariely D. Bracha A. Meier S. (2009). Doing good or doing well? Image motivation and monetary incentives in behaving prosocially. *Am. Econ. Rev.* 99 544–555. 10.1257/aer.99.1.544

[B4] Bem D. J. (1972). Self-perception theory. *Adv. Exp. Soc. Psychol.* 6 1–62. 10.1016/S0065-2601(08)60024-6

[B5] Bolton G. E. Katok E. Zwick R. (1998). Dictator game giving: rules of fairness versus acts of kindness. *Int. J. Game Theory* 27 269–299. 10.1007/s001820050072

[B6] Brandon A. List J. A. Metcalfe R. D. Price M. K. Rundhammer F. (2018). Testing for crowd out in social nudges: evidence from a natural field experiment in the market for electricity. *Proc. Natl. Acad. Sci. U.S.A.* 10.1073/pnas.1802874115 [Epub ahead of print]. 30104369 PMC6431171

[B7] Brandts J. Charness G. (2011). The strategy versus the direct-response method: a first survey of experimental comparisons. *Exp. Econ.* 14 375–398. 10.1007/s10683-011-9272-x

[B8] Clark C. F. Kotchen M. J. Moore M. R. (2003). Internal and external influences on pro-environmental behavior: participation in a green electricity program. *J. Environ. Psychol.* 23 237–246. 10.1016/S0272-4944(02)00105-6

[B9] Clayton S. D. Opotow S. (eds) (2003). *Identity and the Natural Environment: The Psychological Significance of Nature.* Cambridge, MA: MIT Press 10.7551/mitpress/3644.001.0001

[B10] Clot S. Grolleau G. Ibanez L. (2016). Do good deeds make bad people? *Eur. J. Law Econ.* 42 491–513. 10.1007/s10657-014-9441-4

[B11] Cook A. J. Kerr G. N. Moore K. (2002). Attitudes and intentions towards purchasing GM food. *J. Econ. Psychol.* 23 557–572. 10.1016/S0167-4870(02)00117-4

[B12] Cornelissen G. Pandelaere M. Warlop L. Dewitte S. (2008). Positive cueing: promoting sustainable consumer behavior by cueing common environmental behaviors as environmental. *Int. J. Res. Mark.* 25 46–55. 10.1016/j.ijresmar.2007.06.002

[B13] Cracknell J. Miller F. Williams H. (2013). *Passionate Collaboration? Taking the Pulse of the UK Environmental Sector.* Available at: http://www.greenfunders.org/wp-content/uploads/Passionate-Collaboration-Full-Report.pdf [accessed April 22 2017].

[B14] Crump M. J. McDonnell J. V. Gureckis T. M. (2013). Evaluating Amazon’s Mechanical Turk as a tool for experimental behavioral research. *PLoS One* 8:e57410. 10.1371/journal.pone.0057410 23516406 PMC3596391

[B15] d’Adda G. Capraro V. Tavoni M. (2017). Push, don’t nudge: behavioral spillovers and policy instruments. *Econ. Lett.* 154 92–95. 10.1016/j.econlet.2017.02.029

[B16] Davidov E. Schmidt P. Schwartz S. H. (2008). Bringing values back in: the adequacy of the European Social Survey to measure values in 20 countries. *Public Opin. Q.* 72 420–445. 10.1093/poq/nfn035

[B17] Dhar R. Simonson I. (1999). Making complementary choices in consumption episodes: highlighting versus balancing. *J. Mark. Res.* 36 29–44. 10.1177/002224379903600103

[B18] Dolan P. Galizzi M. M. (2015). Like ripples on a pond: behavioral spillovers and their implications for research and policy. *J. Econ. Psychol.* 47 1–16. 10.1016/j.joep.2014.12.003

[B19] Ferraro P. J. Miranda J. J. Price M. K. (2011). The persistence of treatment effects with norm-based policy instruments: evidence from a randomized environmental policy experiment. *Am. Econ. Rev.* 101 318–322. 10.1257/aer.101.3.318

[B20] Festinger L. (1962). *A Theory of Cognitive Dissonance* Vol. 2 Palo Alto, CA: Stanford University Press

[B21] Fielding K. S. McDonald R. Louis W. R. (2008). Theory of planned behaviour, identity and intentions to engage in environmental activism. *J. Environ. Psychol.* 28 318–326. 10.1016/j.jenvp.2008.03.003

[B22] Fischbacher U. Gachter S. (2010). Social preferences, beliefs, and the dynamics of free riding in public goods experiments. *Am. Econ. Rev.* 100 541–556. 10.1257/aer.100.1.541

[B23] Fischbacher U. Gächter S. Fehr E. (2001). Are people conditionally cooperative? Evidence from a public goods experiment. *Econ. Lett.* 71 397–404. 10.1016/S0165-1765(01)00394-9

[B24] Fishbach A. Dhar R. (2005). Goals as excuses or guides: the liberating effect of perceived goal progress on choice. *J. Consum. Res.* 32 370–377. 10.1086/497548

[B25] Fishbach A. Dhar R. Zhang Y. (2006). Subgoals as substitutes or complements: the role of goal accessibility. *J. Pers. Soc. Psychol.* 91 232–242. 10.1037/0022-3514.91.2.232 16881761

[B26] Fishbach A. Zhang Y. Koo M. (2009). The dynamics of self-regulation. *Eur. Rev. Soc. Psychol.* 20 315–344. 10.1080/10463280903275375

[B27] Gallup (2010). *Green Behaviors Common in U.S., but Not Increasing.* Available at: http://www.gallup.com/poll/127292/green-behaviors-common-not-increasing.aspx [accessed April 20 2017].

[B28] Gatersleben B. Murtagh N. Abrahamse W. (2014). Values, identity and pro-environmental behaviour. *Contemp. Soc. Sci.* 9 374–392. 10.1080/21582041.2012.682086

[B29] Ghesla C. Grieder M. Schmitz J. (2018). Nudge for Good? Choice Defaults and Spillover Effects. 10.2139/ssrn.2942744 PMC6379727 30809164

[B30] Gneezy A. Imas A. Brown A. Nelson L. D. Norton M. I. (2012). Paying to be nice: consistency and costly prosocial behavior. *Manag. Sci.* 58 179–187. 10.1287/mnsc.1110.1437

[B31] Goldstein N. J. Cialdini R. B. Griskevicius V. (2008). A room with a viewpoint: using social norms to motivate environmental conservation in hotels. *J. Consum. Res.* 35 472–482. 10.1086/586910

[B32] Guadagno R. E. Asher T. Demaine L. J. Cialdini R. B. (2001). When saying yes leads to saying no: preference for consistency and the reverse foot-in-the-door effect. *Pers. Soc. Psychol. Bull.* 27 859–867. 10.1177/0146167201277008

[B33] Guagnano G. A. Dietz T. Stern P. C. (1994). Willingness to pay for public goods: a test of the contribution model. *Psychol. Sci.* 5 411–415. 10.1111/j.1467-9280.1994.tb00295.x

[B34] Harries T. Rettie R. Studley M. Burchell K. Chambers S. (2013). Is social norms marketing effective? A case study in domestic electricity consumption. *Eur. J. Mark.* 47 1458–1475. 10.1108/EJM-10-2011-0568

[B35] Horton J. J. Rand D. G. Zeckhauser R. J. (2011). The online laboratory: conducting experiments in a real labor market. *Exp. Econ.* 14 399–425. 10.1007/s10683-011-9273-9

[B36] James L. R. Mulaik S. A. Brett J. M. (2006). A tale of two methods. *Organ. Res. Methods* 9 233–244. 10.1177/1094428105285144

[B37] Jordan J. Mullen E. Murnighan J. K. (2011). Striving for the moral self: the effects of recalling past moral actions on future moral behavior. *Pers. Soc. Psychol. Bull.* 37 701–713. 10.1177/0146167211400208 21402752

[B38] Kahneman D. Ritov I. Jacowitz K. E. Grant P. (1993). Stated willingness to pay for public goods: a psychological perspective. *Psychol. Sci.* 4 310–315. 10.1111/j.1467-9280.1993.tb00570.x

[B39] Kant I. (2013). *Moral Law: Groundwork of the Metaphysics of Morals.* Abingdon: Routledge.

[B40] Khan U. Dhar R. (2006). Licensing effect in consumer choice. *J. Mark. Res.* 43 259–266. 10.1509/jmkr.43.2.259

[B41] Klöckner C. A. (2013). “How powerful are moral motives in environmental protection? An integrated model framework,” in *Handbook of Moral Motivation: Theories, Models and Applications* eds Heinrichs K. Oser F. Lovat T. (Rotterdam: Sense).

[B42] Lee Y. K. Chang C. T. (2007). Who gives what to charity? Characteristics affecting donation behavior. *Soc. Behav. Pers.* 35 1173–1180. 10.2224/sbp.2007.35.9.1173

[B43] MacKinnon D. P. Fairchild A. J. Fritz M. S. (2007). Mediation analysis. *Annu. Rev. Psychol.* 58 593–614. 10.1146/annurev.psych.58.110405.085542 16968208 PMC2819368

[B44] Mazar N. Amir O. Ariely D. (2008). The dishonesty of honest people: a theory of self-concept maintenance. *J. Mark. Res.* 45 633–644. 10.1509/jmkr.45.6.633

[B45] Mazar N. Zhong C. B. (2010). Do green products make us better people? *Psychol. Sci.* 21 494–498. 10.1177/0956797610363538 20424089

[B46] Merritt A. C. Effron D. A. Fein S. Savitsky K. K. Tuller D. M. Monin B. (2012). The strategic pursuit of moral credentials. *J. Exp. Soc. Psychol.* 48 774–777. 10.1016/j.jesp.2011.12.017

[B47] Merritt A. C. Effron D. A. Monin B. (2010). Moral self-licensing: when being good frees us to be bad. *Soc. Pers. Psychol. Compass* 4 344–357. 10.1111/j.1751-9004.2010.00263.x

[B48] Miller D. T. Effron D. A. (2010). Psychological license: when it is needed and how it functions. *Adv. Exp. Soc. Psychol.* 43 115–155. 10.1016/S0065-2601(10)43003-8

[B49] Monin B. Miller D. T. (2001). Moral credentials and the expression of prejudice. *J. Pers. Soc. Psychol.* 81 33–43. 10.1037/0022-3514.81.1.33 11474723

[B50] Mukhopadhyay A. Sengupta J. Ramanathan S. (2008). Recalling past temptations: an information-processing perspective on the dynamics of self-control. *J. Consum. Res.* 35 586–599. 10.1086/591105

[B51] Mullen E. Monin B. (2016). Consistency versus licensing effects of past moral behavior. *Annu. Rev. Psychol.* 67 363–385. 10.1146/annurev-psych-010213-115120 26393870

[B52] Nigbur D. Lyons E. Uzzell D. (2010). Attitudes, norms, identity and environmental behaviour: using an expanded theory of planned behaviour to predict participation in a kerbside recycling programme. *Br. J. Soc. Psychol.* 49 259–284. 10.1348/014466609X449395 19486547

[B53] Nolan J. M. Schultz P. W. Cialdini R. B. Goldstein N. J. Griskevicius V. (2008). Normative social influence is underdetected. *Pers. Soc. Psychol. Bull.* 34 913–923. 10.1177/0146167208316691 18550863

[B54] Nomura H. John P. C. Cotterill S. (2011). The use of feedback to enhance environmental outcomes: a randomised controlled trial of a food waste scheme. *Local Environ.* 16 637–653. 10.1080/13549839.2011.586026

[B55] Paolacci G. Chandler J. Ipeirotis P. G. (2010). Running experiments on Amazon mechanical Turk. *Judgm. Decis. Mak.* 5 411–419.

[B56] Peters A. M. van der Werff E. Steg L. (2018). Beyond purchasing: electric vehicle adoption motivation and consistent sustainable energy behaviour in The Netherlands. *Energy Res. Soc. Sci.* 39 234–247. 10.1016/j.erss.2017.10.008

[B57] Pharoah C. (2017). *Top 100 Fundraising Charities Spotlight.* Available at: http://secure.charityfinancials.com/PDF/Top%20100%20Fundraising%20Charities%20rNEW.pdf [accessed April 22 2017].

[B58] Preacher K. J. Hayes A. F. (2008). “Contemporary approaches to assessing mediation in communication research,” in *The Sage Sourcebook of Advanced Data analysis Methods for Communication Research* eds Hayes A. F. Slater M. D. Snyder L. B. (Thousand Oaks, CA: Sage Publications, Inc) 13–54.

[B59] Préget R. Nguyen-Van P. Willinger M. (2016). Who are the voluntary leaders? Experimental evidence from a sequential contribution game. *Theory Decis.* 81 581–599. 10.1007/s11238-016-9550-3

[B60] Sachdeva S. Iliev R. Medin D. L. (2009). Sinning saints and saintly sinners: the paradox of moral self-regulation. *Psychol. Sci.* 20 523–528. 10.1111/j.1467-9280.2009.02326.x 19320857

[B61] Schmitz J. (2018). Temporal dynamics of pro-social behavior - an experimental analysis. *Exp. Econ.* 10.2139/ssrn.2538410 29327424

[B62] Schultz P. W. (1999). Changing behavior with normative feedback interventions: a field experiment on curbside recycling. *Basic Appl. Soc. Psychol.* 21 25–36. 10.1207/s15324834basp2101_3

[B63] Schultz P. W. Nolan J. M. Cialdini R. B. Goldestein N. J. Griskevieius V. (2007). The constructive, deconstructive, and reconstructive power of social norms. *J. Psychol. Sci.* 18 429–434. 10.1111/j.1467-9280.2007.01917.x 17576283

[B64] Shah J. Y. Friedman R. Kruglanski A. W. (2002). Forgetting all else: on the antecedents and consequences of goal shielding. *J. Pers. Soc. Psychol.* 83 1261–1280. 10.1037/0022-3514.83.6.1261 12500810

[B65] Shrout P. E. Bolger N. (2002). Mediation in experimental and nonexperimental studies: new procedures and recommendations. *Psychol. Methods* 7 422–445. 10.1037/1082-989X.7.4.422 12530702

[B66] Steg L. Bolderdijk J. W. Keizer K. Perlaviciute G. (2014). An integrated framework for encouraging pro-environmental behaviour: the role of values, situational factors and goals. *J. Environ. Psychol.* 38 104–115. 10.1016/j.jenvp.2014.01.002

[B67] Strahilevitz M. Myers J. G. (1998). Donations to charity as purchase incentives: how well they work may depend on what you are trying to sell. *J. Consum. Res.* 24 434–446. 10.1086/209519

[B68] Strauss B. (2017). *The Top 10 Wildlife Conservation Organizations.* Available at: https://www.thoughtco.com/top-wildlife-conservation-organizations-4088567 [accessed April 22 2017].

[B69] Suri S. Watts D. J. (2011). Cooperation and contagion in web-based, networked public goods experiments. *PLoS One* 6:e16836. 10.1371/journal.pone.0016836 21412431 PMC3055889

[B70] Susewind M. Hoelzl E. (2014). A matter of perspective: why past moral behavior can sometimes encourage and other times discourage future moral striving. *J. Appl. Soc. Psychol.* 44 201–209. 10.1111/jasp.12214

[B71] Thøgersen J. (1996). Recycling and morality: a critical review of the literature. *Environ. Behav.* 28 536—558. 10.1177/0013916596284006

[B72] Thøgersen J. (2004). A cognitive dissonance interpretation of consistencies and inconsistencies in environmentally responsible behavior. *J. Environ. Psychol.* 24 93–103. 10.1016/S0272-4944(03)00039-2

[B73] Thøgersen J. Crompton T. (2009). Simple and painless? The limitations of spillover in environmental campaigning. *J. Consum. Policy* 32 141–163. 10.1007/s10603-009-9101-1

[B74] Thøgersen J. Ölander F. (2003). Spillover of environment-friendly consumer behaviour. *J. Environ. Psychol.* 23 225–236. 10.1016/S0272-4944(03)00018-5

[B75] Tiefenbeck V. Staake T. Roth K. Sachs O. (2013). For better or for worse? Empirical evidence of moral licensing in a behavioral energy conservation campaign. *Energy Policy* 57 160–171. 10.1016/j.enpol.2013.01.021

[B76] Toelch U. Bruce M. J. Meeus M. T. Reader S. M. (2011). Social performance cues induce behavioral flexibility in humans. *Front. Psychol.* 2:160. 10.3389/fpsyg.2011.00160 21811477 PMC3139953

[B77] Truelove H. B. Carrico A. R. Weber E. U. Raimi K. T. Vandenbergh M. P. (2014). Positive and negative spillover of pro-environmental behavior: an integrative review and theoretical framework. *Glob. Environ. Change* 29 127–138. 10.1016/j.gloenvcha.2014.09.004

[B78] Van der Werff E. Steg L. Keizer K. (2013a). It is a moral issue: the relationship between environmental self-identity, obligation-based intrinsic motivation and pro-environmental behaviour. *Glob. Environ. Change* 23 1258–1265. 10.1016/j.gloenvcha.2013.07.018

[B79] Van der Werff E. Steg L. Keizer K. (2013b). The value of environmental self-identity: the relationship between biospheric values, environmental self-identity and environmental preferences, intentions and behaviour. *J. Environ. Psychol.* 34 55–63. 10.1016/j.jenvp.2012.12.006

[B80] Van der Werff E. Steg L. Keizer K. (2014a). Follow the signal: when past pro-environmental actions signal who you are. *J. Environ. Psychol.* 40 273–282. 10.1016/j.jenvp.2014.07.004

[B81] Van der Werff E. Steg L. Keizer K. (2014b). I am what I am, by looking past the present: the influence of biospheric values and past behavior on environmental self-identity. *Environ. Behav.* 46 626–657. 10.1177/0013916512475209

[B82] Whitmarsh L. O’Neill S. (2010). Green identity, green living? The role of pro-environmental self-identity in determining consistency across diverse pro-environmental behaviours. *J. Environ. Psychol.* 30 305–314. 10.1016/j.jenvp.2010.01.003

[B83] Wiltermuth S. S. Monin B. Chow R. M. (2010). The orthogonality of praise and condemnation in moral judgment. *Soc. Psychol. Personal. Sci.* 1 302–310. 10.1177/1948550610363162

[B84] Zhong C.-B. Liljenquist K. Cain D. M. (2009). “Moral self-regulation: licensing and compensation,” in *Psychological Perspectives on Ethical Behavior and Decision Making* ed. De Cremer D. (Charlotte, NC: Information Age Publishing) 75–89.

[B85] Zhong C. B. Strejcek B. Sivanathan N. (2010). A clean self can render harsh moral judgment. *J. Exp. Soc. Psychol.* 46 859–862. 10.1016/j.jesp.2010.04.003

