# Supplementary material for: On the Use of Nudges to Affect Spillovers in Environmental Behaviors
Source: Front Psychol. 2019 Jan 29;10:61. doi: 10.3389/fpsyg.2019.00061 (PMC6362870; doi:10.3389/fpsyg.2019.00061)
Supplement: Supplementary file 1 [file Table_1.DOCX]

Supplementary Material

On the use of nudges to affect spillovers in environmental behaviors

Valeria Fanghella*, Giovanna d’Adda, Massimo Tavoni

*** Correspondence:** valeria.fanghella@unitn.it

# Supplementary material

## Pilot test

1. Which is your gender?
   1. Male
   2. Female
2. Which is your year of birth?
3. What is the highest educational level that you have attained?
   1. Primary school or lower
   2. Secondary school
   3. Bachelor’s degree
   4. Postgraduate degree
4. Here is a list of monthly incomes. In what group your household is, counting all wages, salaries, pensions and other incomes that come in, net of taxes and other deductions?
   1. Less than £ 500
   2. From £ 500 to 1,000
   3. From £ 1,000 to 2,000
   4. From £ 2,000 to3,000
   5. From £ 3,000 to5,000
   6. From £ 5,000 to 7,000
   7. From £ 7,000 to10,000
   8. More than £ 10,000

Since everyone has different ideas about supporting environmental organisations, we are using this survey to understand individuals' behaviour in case they have the chance to choose whether to support or not one of them. Best known as the world’s leading conservation body, WWF is active in safeguarding the natural world, tackling the global threat of climate change and helping people to change the way they live.

1. Do you think that supporting an environmental charity like WWF help contribute to address environmental issues?
   1. Strongly agree
   2. Somewhat agree
   3. Neither agree nor disagree
   4. Somewhat disagree
   5. Strongly disagree
2. If you had the opportunity to donate part of your participation fee of this survey (£ 0.5) to WWF, how much would you donate?

Note: we will NOT subtract the selected amount from your fee, it is a hypothetical decision.

Donation in £:

## Study 1

1. Which is your gender?
   1. Male
   2. Female
2. Which is your year of birth?
3. What is the highest educational level that you have attained?
4. Primary school or lower
5. Secondary school
6. Bachelor’s degree
7. Postgraduate degree
8. Here is a list of monthly incomes. In what group your household is, counting all wages, salaries, pensions and other incomes that come in, net of taxes and other deductions?
9. Less than £ 500
10. From £ 500 to 1,000
11. From £ 1,000 to 2,000
12. From £ 2,000 to3,000
13. From £ 3,000 to5,000
14. From £ 5,000 to 7,000
15. From £ 7,000 to10,000
16. More than £ 10,000
17. (*Identity priming*) Which of the following environmental activities do you perform? Please indicate how often do you perform them.

|  | 1 | 2 | 3 | 4 | 5 |
| --- | --- | --- | --- | --- | --- |
| I turn off the lights when no one is in the room |  |  |  |  |  |
| I do not throw litter on the street |  |  |  |  |  |
| I recycle newspapers, glass, aluminium, motor oil, or other items |  |  |  |  |  |
| I turn off electrical appliances (to save energy) |  |  |  |  |  |
| I move around by bike and/or public transportation |  |  |  |  |  |
| I buy a less polluting product if there is a choice in the shop |  |  |  |  |  |
| I use reusable shopping bags at grocery stores instead of the standard plastic or paper bags |  |  |  |  |  |
| I leave a clean spot after a picnic |  |  |  |  |  |

1. (*Control*) Which of the following activities do you perform? Please indicate how often do you perform them.

|  | 1 | 2 | 3 | 4 | 5 |
| --- | --- | --- | --- | --- | --- |
| I read the newspaper |  |  |  |  |  |
| I go to the theatre to watch drama |  |  |  |  |  |
| I spend my weekend with my family and/or my friends |  |  |  |  |  |
| I play multi-player online videogames |  |  |  |  |  |
| I follow the precepts of my religion, if any |  |  |  |  |  |
| I go to pubs/bars/clubs |  |  |  |  |  |
| I share my experiences on social networks |  |  |  |  |  |
| I listen to music on the radio |  |  |  |  |  |

1. Please indicate to what extent you agree with the following statements:

|  | 1 | 2 | 3 | 4 | 5 | 6 | 7 |
| --- | --- | --- | --- | --- | --- | --- | --- |
| Acting pro-environmentally is an important part of who I am |  |  |  |  |  |  |  |
| I am the type of person who acts in an environmentally-friendly way |  |  |  |  |  |  |  |
| I see myself as an environmentally-friendly person |  |  |  |  |  |  |  |

1. (*Social information*) Everyone has different ideas about supporting environmental causes. Especially, we are using this survey to understand individuals' attitudes toward environmental organisations.

As part of this survey you will be asked whether you want to make a donation to WWF UK. Best known as the world’s leading conservation body, WWF is active in safeguarding the natural world, tackling the global threat of climate change and helping people to change the way they live. Last week, we conducted a similar survey on Prolific: participants were willing to donate on average 40% of their bonus to WWF UK. Would you like to donate part of your participation bonus to WWF UK?

Please enter a donation amount between 0£ and 1£. The donation will be subtracted from your bonus payment of 1£. We will send you a proof of donation by email.

Donation in £: …

1. (*Control*) Everyone has different ideas about supporting environmental causes. Especially, we are using this survey to understand individuals' attitudes toward environmental organisations.

As part of this survey you will be asked whether you want to make a donation to WWF UK. Best known as the world’s leading conservation body, WWF is active in safeguarding the natural world, tackling the global threat of climate change and helping people to change the way they live. Would you like to donate part of your participation bonus to WWF UK?

Please enter a donation amount between 0£ and 1£. The donation will be subtracted from your bonus payment of 1£. We will send you a proof of donation by email.

Donation in £: ...

1. What is your preferred way to support social and environmental causes? Select all that apply.
2. Volunteering/active membership in a charity
3. Donating money to a charity
4. Fundraising for a charity (e.g., organising a marathon)
5. Sign a petition
6. None
7. Which of the following best describes how often you give to charity?
8. Weekly
9. Monthly
10. From time-to-time
11. Rarely
12. Never
13. Which of the following causes do you usually support? Select all that apply.
14. Animal welfare
15. Homeless and housing
16. Overseas aid and disaster relief
17. Hospital and hospices
18. Physical and mental health care
19. Environment
20. Religious organisation
21. Other
22. None
23. When was the last time you donated to/volunteering for an environmental organisation?
24. Within the last 6 months
25. Within the last year
26. Between 2 and 5 years ago
27. Never
28. Now we will briefly describe some people. Would you please indicate for each description whether that person is very much like you, like you, somewhat like you, not like you, or not at all like you?

|  | 1 | 2 | 3 | 4 | 5 | 6 |
| --- | --- | --- | --- | --- | --- | --- |
| It is important to this person to be rich; to have a lot of money and expensive things. |  |  |  |  |  |  |
| It is important that every person in the world is treated equally; everyone should have equal opportunities in life. |  |  |  |  |  |  |
| It is important to this person to show his/her abilities; to have people admire what this person does. |  |  |  |  |  |  |
| It is important to this person to listen to people who are different from him/her; even in case of disagreement, this person wants to understand them. |  |  |  |  |  |  |
| It is important to this person to have a good time; to “spoil” oneself. |  |  |  |  |  |  |
| It is important to this person to help the people nearby; take care if their well-being. |  |  |  |  |  |  |
| Being very successful is important to this person; to have people recognize one’s achievements. |  |  |  |  |  |  |
| It is important to this person to get respect from others; to make people do what this person says. |  |  |  |  |  |  |
| It is important to this person to be loyal to friends; to devote to people close to him/her. |  |  |  |  |  |  |
| This person strongly believes that people should care for nature. Looking after the environment is important to this person. |  |  |  |  |  |  |
| This person seeks every chance to have fun; it is important to this person to do things that give pleasure. |  |  |  |  |  |  |

1. Please indicate to what extent you agree with the following statements

|  | 1 | 2 | 3 | 4 | 5 | 6 | 7 |
| --- | --- | --- | --- | --- | --- | --- | --- |
| I feel morally obliged to act in an environmentally-friendly manner |  |  |  |  |  |  |  |
| - I worry about the environmental impact of CO2 emissions caused by human activities |  |  |  |  |  |  |  |
| - I think it is useful to reduce CO2 emissions to reduce environmental problems |  |  |  |  |  |  |  |
| I would feel guilty if I did not act in an environmentally-friendly manner |  |  |  |  |  |  |  |
| - Human activities cause serious environmental problems, such as climate change |  |  |  |  |  |  |  |
| I would be a better person if I would act in an environmentally-friendly manner |  |  |  |  |  |  |  |
| - I think I can contribute to reduce environmental problems with my behaviours |  |  |  |  |  |  |  |

1. Listed below are statements about the relationship between humans and the environment. For each one, please indicate to what extend you agree with them.

|  | 1 | 2 | 3 | 4 | 5 |
| --- | --- | --- | --- | --- | --- |
| We are approaching the limit of the number of people the earth can support |  |  |  |  |  |
| Humans have the right to modify the natural environment to suit their needs |  |  |  |  |  |
| When humans interfere with nature it often produces disastrous consequences |  |  |  |  |  |
| Human ingenuity will insure that we do NOT make the earth unliveable |  |  |  |  |  |
| Humans are severely abusing the environment |  |  |  |  |  |
| The earth has plenty of natural resources if we just learn how to develop them |  |  |  |  |  |
| Plants and animals have as much right as humans to exist |  |  |  |  |  |
| The balance of nature is strong enough to cope with the impacts of modern industrial nations |  |  |  |  |  |
| Despite our special abilities humans are still subject to the laws of nature |  |  |  |  |  |
| The so-called ‘ecological-crisis’ facing humankind has been greatly exaggerated |  |  |  |  |  |
| The earth is like a spaceship with very limited room and resources |  |  |  |  |  |
| Humans were meant to rule over the rest of nature |  |  |  |  |  |
| The balance of nature is very delicate and easily upset |  |  |  |  |  |
| Humans will eventually learn enough about how nature works to be able to control it |  |  |  |  |  |
| If things continue on their present course, we will soon experience a major ecological catastrophe |  |  |  |  |  |

## Study 2

Payment of the survey

You will receive £ 1 as a partecipation reward. In addition, you will receive a bonus of up to £ 1 depending on your decisions.

You have to make two decisions. We will pick one of the two decisions at random, and pay you depending on what you have chosen in that one. We will send a proof of donation after the survey is ended.

Decisions you will make:

Unconditional donation: You have to decide how much of your bonus payment of £ 1 you want to donate to an environmental charity. Example: You have been randomly assigned to the unconditional donation group. You have indicated that your unconditional donation is £ 0.7. Your donation will be £ 0.7 and your bonus payment £ 1-0.7= £ 0.3.

Conditional donation: You have to decide how much of your bonus payment of £ 1 you want to donate to an environmental charity, given a series of donations of the other participants to this survey. Example: You have been randomly assigned to the conditional donation group. The average donation from the participants assigned to the unconditional donation is £ 0.2. If you have indicated that you would donate £ 0.7 if the others donate £ 0.2 on average. Your donation will be £ 0.7, and your bonus payment £ 1-0.7= £ 0.3.

1. Which is your gender?
   1. Male
   2. Female
2. Which is your year of birth?
3. What is the highest educational level that you have attained?
4. Primary school or lower
5. Secondary school
6. Bachelor’s degree
7. Postgraduate degree
8. Here is a list of monthly incomes. In what group your household is, counting all wages, salaries, pensions and other incomes that come in, net of taxes and other deductions?
9. Less than £ 500
10. From £ 500 to 1,000
11. From £ 1,000 to 2,000
12. From £ 2,000 to3,000
13. From £ 3,000 to5,000
14. From £ 5,000 to 7,000
15. From £ 7,000 to10,000
16. More than £ 10,000
17. (*Identity priming*) Which of the following environmental activities do you perform? Please indicate how often do you perform them.

|  | 1 | 2 | 3 | 4 | 5 |
| --- | --- | --- | --- | --- | --- |
| I turn off the lights when no one is in the room |  |  |  |  |  |
| I do not throw litter on the street |  |  |  |  |  |
| I recycle newspapers, glass, aluminium, motor oil, or other items |  |  |  |  |  |
| I turn off electrical appliances (to save energy) |  |  |  |  |  |
| I move around by bike and/or public transportation |  |  |  |  |  |
| I buy a less polluting product if there is a choice in the shop |  |  |  |  |  |
| I use reusable shopping bags at grocery stores instead of the standard plastic or paper bags |  |  |  |  |  |
| I leave a clean spot after a picnic |  |  |  |  |  |

1. (*Control*) Which of the following activities do you perform? Please indicate how often do you perform them.

|  | 1 | 2 | 3 | 4 | 5 |
| --- | --- | --- | --- | --- | --- |
| I read the newspaper |  |  |  |  |  |
| I go to the theatre to watch drama |  |  |  |  |  |
| I spend my weekend with my family and/or my friends |  |  |  |  |  |
| I play multi-player online videogames |  |  |  |  |  |
| I follow the precepts of my religion, if any |  |  |  |  |  |
| I go to pubs/bars/clubs |  |  |  |  |  |
| I share my experiences on social networks |  |  |  |  |  |
| I listen to music on the radio |  |  |  |  |  |

1. (*Goal commitment*) Please think about three reasons why you perform these activities and use the following boxes to write them.
2. Please indicate to what extent you agree with the following statements:

|  | 1 | 2 | 3 | 4 | 5 | 6 | 7 |
| --- | --- | --- | --- | --- | --- | --- | --- |
| Acting pro-environmentally is an important part of who I am |  |  |  |  |  |  |  |
| I am the type of person who acts in an environmentally-friendly way |  |  |  |  |  |  |  |
| I see myself as an environmentally-friendly person |  |  |  |  |  |  |  |

1. Would you like to donate part of your participation bonus to WWF UK?

- Unconditional donation: Please enter a donation between £ 0 and £ 1. If this question is selected, the donation will be subtracted from your bonus payment of £ 1 as explained at the beginning of the survey. We will send you a proof of donation by email.

Donation:….

- Conditional donation: Given the possible donations of other participants, would you like to donate part of your participation bonus to WWF UK?

Please enter in each cell a donation between £ 0 and £ 1. If this question is selected, the donation will be subtracted from your bonus payment of £ 1 as explained at the beginning of the survey. We will send you a proof of donation by email.

| Average donation of other participants | Your conditional donation |
| --- | --- |
| 0 |  |
| 0.1 |  |
| 0.2 |  |
| 0.3 |  |
| 0.4 |  |
| 0.5 |  |
| 0.6 |  |
| 0.7 |  |
| 0.8 |  |
| 0.9 |  |
| 1 |  |

1. Now we will briefly describe some people. Would you please indicate for each description whether that person is very much like you, like you, somewhat like you, not like you, or not at all like you?

|  | 1 | 2 | 3 | 4 | 5 | 6 |
| --- | --- | --- | --- | --- | --- | --- |
| It is important to this person to be rich; to have a lot of money and expensive things. |  |  |  |  |  |  |
| It is important that every person in the world is treated equally; everyone should have equal opportunities in life. |  |  |  |  |  |  |
| It is important to this person to show his/her abilities; to have people admire what this person does. |  |  |  |  |  |  |
| It is important to this person to listen to people who are different from him/her; even in case of disagreement, this person wants to understand them. |  |  |  |  |  |  |
| It is important to this person to have a good time; to “spoil” oneself. |  |  |  |  |  |  |
| It is important to this person to help the people nearby; take care if their well-being. |  |  |  |  |  |  |
| Being very successful is important to this person; to have people recognize one’s achievements. |  |  |  |  |  |  |
| It is important to this person to get respect from others; to make people do what this person says. |  |  |  |  |  |  |
| It is important to this person to be loyal to friends; to devote to people close to him/her. |  |  |  |  |  |  |
| This person strongly believes that people should care for nature. Looking after the environment is important to this person. |  |  |  |  |  |  |
| This person seeks every chance to have fun; it is important to this person to do things that give pleasure. |  |  |  |  |  |  |

1. Please indicate to what extent you agree with the following statements

|  | 1 | 2 | 3 | 4 | 5 | 6 | 7 |
| --- | --- | --- | --- | --- | --- | --- | --- |
| I feel morally obliged to act in an environmentally-friendly manner |  |  |  |  |  |  |  |
| - I worry about the environmental impact of CO2 emissions caused by human activities |  |  |  |  |  |  |  |
| - I think it is useful to reduce CO2 emissions to reduce environmental problems |  |  |  |  |  |  |  |
| I would feel guilty if I did not act in an environmentally-friendly manner |  |  |  |  |  |  |  |
| - Human activities cause serious environmental problems, such as climate change |  |  |  |  |  |  |  |
| I would be a better person if I would act in an environmentally-friendly manner |  |  |  |  |  |  |  |
| - I think I can contribute to reduce environmental problems with my behaviours |  |  |  |  |  |  |  |

1. Listed below are statements about the relationship between humans and the environment. For each one, please indicate to what extend you agree with them.

|  | 1 | 2 | 3 | 4 | 5 |
| --- | --- | --- | --- | --- | --- |
| We are approaching the limit of the number of people the earth can support |  |  |  |  |  |
| Humans have the right to modify the natural environment to suit their needs |  |  |  |  |  |
| When humans interfere with nature it often produces disastrous consequences |  |  |  |  |  |
| Human ingenuity will insure that we do NOT make the earth unliveable |  |  |  |  |  |
| Humans are severely abusing the environment |  |  |  |  |  |
| The earth has plenty of natural resources if we just learn how to develop them |  |  |  |  |  |
| Plants and animals have as much right as humans to exist |  |  |  |  |  |
| The balance of nature is strong enough to cope with the impacts of modern industrial nations |  |  |  |  |  |
| Despite our special abilities humans are still subject to the laws of nature |  |  |  |  |  |
| The so-called ‘ecological-crisis’ facing humankind has been greatly exaggerated |  |  |  |  |  |
| The earth is like a spaceship with very limited room and resources |  |  |  |  |  |
| Humans were meant to rule over the rest of nature |  |  |  |  |  |
| The balance of nature is very delicate and easily upset |  |  |  |  |  |
| Humans will eventually learn enough about how nature works to be able to control it |  |  |  |  |  |
| If things continue on their present course, we will soon experience a major ecological catastrophe |  |  |  |  |  |

# Supplementary tables

Table 1. Demographic characteristics of the sample of Study 1 per experimental condition

|  | IP | | SI | | IP – SI | | Control | |
| --- | --- | --- | --- | --- | --- | --- | --- | --- |
|  | M | SD | M | SD | M | SD | M | SD |
|  |  |  |  |  |  |  |  |  |
| Gender (percentage female) | 0.463 | 0.501 | 0.411 | 0.494 | 0.442 | 0.499 | 0.466 | 0.501 |
| Year of birth | 1985.726 | 9.522 | 1986.484 | 9.895 | 1986.106 | 9.862 | 1986.010 | 10.526 |
| Schooling |  |  |  |  |  |  |  |  |
| Schooling: primary or lower | 0.021 | 0.144 | 0.021 | 0.144 | 0.010 | 0.098 | 0.000 | 0.000 |
| Schooling: secondary school | 0.337^1^ | 0.475 | 0.389 | 0.490 | 0.500^1^ | 0.502 | 0.456 | 0.500 |
| Schooling: bachelor’s degree | 0.389 | 0.490 | 0.474^a^ | 0.502 | 0.337^a^ | 0.475 | 0.398 | 0.492 |
| Schooling: postgraduate degree | 0.253^b,2,c^ | 0.437 | 0.116^2^ | 0.322 | 0.154^b^ | 0.363 | 0.146^c^ | 0.354 |
| Income |  |  |  |  |  |  |  |  |
| Household income: less than 500 € | 0.074 | 0.263 | 0.063 | 0.244 | 0.029 | 0.168 | 0.078 | 0.269 |
| Household income: between 500 and 1,000 € | 0.147 | 0.356 | 0.105 | 0.309 | 0.183 | 0.388 | 0.126 | 0.334 |
| Household income: between 1,000 and 2,000 € | 0.284 | 0.453 | 0.389^3^ | 0.502 | 0.250^3^ | 0.435 | 0.320 | 0.469 |
| Household income: between 2,000 and 3,000 € | 0.158 | 0.367 | 0.189 | 0.394 | 0.231 | 0.373 | 0.165 | 0.373 |
| Household income: between 3,000 and 5,000 € | 0.137 | 0.345 | 0.116 | 0.322 | 0.135 | 0.343 | 0.175 | 0.382 |
| Household income: between 5,000 and 7,000 € | 0.074^d^ | 0.263 | 0.042 | 0.202 | 0.058 | 0.234 | 0.019^d^ | 0.139 |
| Household income: between 7,000 and 10,000 € | 0.042 | 0.202 | 0.053 | 0.224 | 0.029 | 0.168 | 0.019 | 0.139 |
| Household income: more than 10,000 € | 0.084 | 0.279 | 0.042 | 0.202 | 0.087 | 0.283 | 0.097 | 0.297 |
| No. of observations | 95 | | 95 | | 104 | | 103 | |

Note: IP denotes the identity priming treatment; SI denotes the social information treatment. Letters and numbers represent the significance of pairwise comparisons per experimental condition with T-test with different variances, two tails. Same letter in the row represents a significant difference with p<.10; same number p<.05.

Table 2. Heterogeneous effects of identity priming in Study 1

|  | (1)  Identity | | | (2)  Average  donation | | | | (3)  Extensive  margin | | | | (4)  Intensive  margin | | |
| --- | --- | --- | --- | --- | --- | --- | --- | --- | --- | --- | --- | --- | --- | --- |
|  | B | SE(B) | | B | | SE(B) | | B | | SE(B) | | B | | SE(B) |
|  |  |  | |  | |  | |  | |  | |  | |  |
| IP | -0.199* | 0.119 | | -0.139** | | 0.058 | | -0.692* | | 0.366 | | -0.174* | | 0.093 |
| SI |  |  | | 0.038 | | 0.051 | | 0.427 | | 0.296 | | -0.057 | | 0.069 |
| IP*SI |  |  | | 0.049 | | 0.072 | | 0.432 | | 0.428 | | 0.035 | | 0.101 |
| IP*High | 0.722*** | 0.139 | | 0.100* | | 0.052 | | 0.547* | | 0.317 | | 0.102 | | 0.074 |
| Univ | 0.717*** | 0.073 | | 0.131*** | | 0.028 | | 0.768*** | | 0.176 | | 0.090** | | 0.043 |
| Const | 2.304*** | 0.302 | | -0.253** | | 0.115 | | -3.493*** | | 0.743 | | 0.293 | | 0.187 |
| Obs | 397 |  | | 397 | |  | | 397 | |  | | 177 | |  |
| R^2 | 0.295 |  | | 0.090 | |  | |  | |  | | 0.062 | |  |
| Adj R^2 | 0.290 |  | | 0.078 | |  | |  | |  | | 0.034 | |  |
| Log Likelihood |  | |  | |  | |  | | -253.086 | |  | |  | |
| Akaike Inf. Crit. |  | |  | |  | |  | | 518.171 | |  | |  | |
| F | 54.78*** | | | 7.707*** | | | |  | | | | 2.252* | | |

Note: Linear regression (Columns 1, 2 and 4). Logit regression (Column 3). IP denotes the identity priming treatment, SI denotes the social information treatment, High is a dummy equal to 1 for the *High frequency* group, Univ denotes universalistic values. Standard errors reported in the SE(B) columns. * significant at 10%; ** significant at 5%; *** significant at 1%.

Table 3. Demographic characteristics of the sample of Study 2 per experimental condition

|  | IP | | IP – GC | | Control | |
| --- | --- | --- | --- | --- | --- | --- |
|  | M | SD | M | SD | M | SD |
|  |  |  |  |  |  |  |
| Gender (percentage female) | 0.500 | 0.502 | 0.532 | 0.501 | 0.553 | 0.499 |
| Year of birth | 1981.649^a,2^ | 11.232 | 1984.135^a^ | 11.353 | 1984.416^2^ | 11.223 |
| Schooling |  |  |  |  |  |  |
| Schooling: primary or lower | 0.006 | 0.081 | 0.006 | 0.080 | 0.006 | 0.079 |
| Schooling: secondary school | 0.383 | 0.488 | 0.436 | 0.497 | 0.404 | 0.492 |
| Schooling: bachelor’s degree | 0.422 | 0.496 | 0.391 | 0.490 | 0.397 | 0.491 |
| Schooling: postgraduate degree | 0.188 | 0.392 | 0.167 | 0.374 | 0.193 | 0.396 |
| Income |  |  |  |  |  |  |
| Household income: less than 500 € | 0.032^b^ | 0.178 | 0.058 | 0.234 | 0.075^b^ | 0.264 |
| Household income: between 500 and 1,000 € | 0.110 | .0314 | 0.109 | 0.313 | 0.137 | 0.344 |
| Household income: between 1,000 and 2,000 € | 0.253 | 0.436 | 0.308^c^ | 0.463 | 0.217^c^ | 0.414 |
| Household income: between 2,000 and 3,000 € | 0.234 | 0.424 | 0.167 | 0.374 | 0.212 | 0.409 |
| Household income: between 3,000 and 5,000 € | 0.123 | 0.323 | 0.167 | 0.374 | 0.156 | 0.363 |
| Household income: between 5,000 and 7,000 € | 0.045 | 0.209 | 0.058 | 0.234 | 0.037 | 0.190 |
| Household income: between 7,000 and 10,000 € | 0.058^2^ | 0.235 | 0.013^2^ | 0.113 | 0.025 | 0.156 |
| Household income: more than 10,000 € | 0.143 | 0.351 | 0.122 | 0.323 | 0.143 | 0.351 |
| No. of observations | 156 | | 154 | | 161 | |

Note: IP denotes the identity priming treatment; GC denotes the goal commitment treatment. Letters and numbers represent the significance of pairwise comparisons per experimental condition with T-test with different variances, two tails. Same letter in the row represents a significant difference with p<.10; same number p<.05.

Table 4. Heterogeneous effects of identity priming and goal commitment in Study 2

|  | (1)  Identity | | | (2)  Average  donation | | | | (3)  Extensive  margin | | | | | (4)  Intensive  margin | | | |
| --- | --- | --- | --- | --- | --- | --- | --- | --- | --- | --- | --- | --- | --- | --- | --- | --- |
|  | B | SE(B) | | B | | SE(B) | | B | | SE(B) | | | B | | | SE(B) |
|  |  |  | |  | |  | |  | | |  | |  | |  | |
| IP | 0.129 | 0.102 | | -0.043 | | 0.049 | | -0.199 | | | 0.308 | | -0.030 | | 0.053 | |
| IP-GC |  |  | | -0.094* | | 0.048 | | -0.231 | | | 0.307 | | -0.087* | | 0.053 | |
| IP*High | 0.432*** | 0.104 | | 0.063 | | 0.057 | | 0.476 | | | 0.383 | | 0.029 | | 0.059 | |
| IP-GC*High |  |  | | 0.123** | | 0.056 | | 0.345 | | | 0.366 | | 0.113* | | 0.059 | |
| Univ | 0.836*** | 0.063 | | 0.163*** | | 0.024 | | 0.906*** | | | 0.166 | | 0.100*** | | 0.028 | |
| Const | 1.712*** | 0.264 | | -0.246** | | 0.102 | | -2.682*** | | | 0.670 | | 0.155 | | 0.122 | |
| Obs | 471 |  | | 471 | |  | | 471 | | |  | | 334 | |  | |
| R^2 | 0.339 |  | | 0.112 | |  | |  | | |  | | 0.060 | |  | |
| Adj R^2 | 0.335 |  | | 0.102 | |  | |  | | |  | | 0.045 | |  | |
| Log Likelihood |  | |  | |  | |  | | -264.331 | | |  | |  | | |
| Akaike Inf. Crit. |  | |  | |  | |  | | 540.662 | | |  | |  | | |
| F | 79.8*** | | | 11.71*** | | | |  | | | | | 4.155*** | | | |

Note: Linear regression (Columns 1, 2 and 4). Logit regression (Column 3). IP denotes the identity priming treatment, GC denotes the goal commitment treatment, High is a dummy equal to 1 for the *High frequency* group, Univ denotes universalistic values. Standard errors reported in the SE(B) columns. * significant at 10%; ** significant at 5%; *** significant at 1%.
